# Plastics to fertilizer: A polymer system based on isosorbide as a monomer, plasticizer, and fertilizer

**DOI:** 10.1038/s41598-026-63638-1

**Published:** 2026-08-18

**Authors:** Shunsuke Fujimata, Yoshiki Tokonami, Raj Kishan Agrahari, Toyokazu Tsutsuba, Kazuaki Rikiyama, Norio Tomotsu, Tatsuo Taniguchi, Takehiro Kamiya, Mizuhiko Nishida, Daisuke Aoki

**Affiliations:** 1 Department of Applied Chemistry and Biotechnology, Graduate School of Engineering, Chiba University, 1-33 Yayoi-cho, Inage-ku, Chiba-shi, 263-8522 Chiba Japan; 2 Field Science Center, Graduate School of Agricultural Science, Tohoku University, 232- 3, Yomogida, Naruko Onsen, Osaki, 989-6711 Miyagi Japan; 3 The Laboratory of Plant Nutrition and Fertilizers, Graduate School of Agricultural and Life Sciences, The University of Tokyo, 1-1-1, Yayoi, Bunkyo-ku, Tokyo, 113-8657 Japan; 4 Research Center for Space Agriculture and Horticulture, Chiba University, 648 Matsudo, Matsudo-shi, 271-8510 Chiba Japan

**Keywords:** Chemistry, Environmental sciences, Materials science

## Abstract

Plastics have long been designed as durable materials with limited consideration of their fate after use. In contrast, a polymer system that enables end-of-life plastics to no longer to be discarded but instead to be transformed into fertilizers that actively benefit ecosystems, would be very desirable indeed. Such a concept may offer a new direction for polymer design and expand the potential roles of plastic materials. In our previous studies, we demonstrated that isosorbide-based polycarbonates (PIC) can be converted into fertilizer-compatible components through ammonolysis after use. Building on this concept, the present study introduces dual-functional isosorbide-based plasticizers that simultaneously reduce the brittleness of PIC while also being convertible into fertilizer-compatible components through ammonolysis after use. The effectiveness of the degradation products obtained from this plastic system, composed of PIC and isosorbide-based plasticizers, as fertilizers was validated through the growth of both a model plant and an edible vegetable.

**Supplementary Information:**

The online version contains supplementary material available at 10.1038/s41598-026-63638-1.

## Introduction

We are at a turning point in the history of plastics, which have enriched our lives and supported industry since their invention. The era of simply using plastics and discarding them is rapidly coming to an end, and efforts are now focused on finding solutions for plastic waste that address issues with end-of-life management and minimize negative environmental impact.^1,2^ In this regard, plastic-use reduction and recycling remain the currently dominant strategies^3–14^, yet these solutions are inherently passive and offer no active environmental benefits. If polymer systems capable of being converted into environmentally beneficial fertilizers after their service life can be realized, they may offer a new direction for polymer design and broaden the functional concept of plastic materials.

Here, we introduce a polymer system based on isosorbide (ISB), which we believe will generate great interest in the field of polymer chemistry. ISB is a type of bio-based monomer, an artificial substance synthesized from glucose.^15,16^ Bio-based monomers are made from renewable resources, thus reducing the use of fossil fuels and lowering the impact on the environment.^17^ Bio-based monomers can be expected to have a stable feedstock and therefore supply in the long term, which will address potential problems associated with resource depletion. ISB in particular is widely used as a building block for polymers due to its biodegradability^18^ and rigid structure.^19–25^ Recently, we have demonstrated that polycarbonate made of isosorbide (PIC) is decomposed into ISB and urea following treatment with aqueous ammonia, both of which can be directly used as a fertilizer after having previously being used in plastics.^26–29^ At first, this fertilizer effect was thought to only be due to the urea contained in the decomposition products, but it was subsequently shown that the combination of urea and ISB does indeed have a positive synergistic effect on plant growth.

**Fig. 1:**
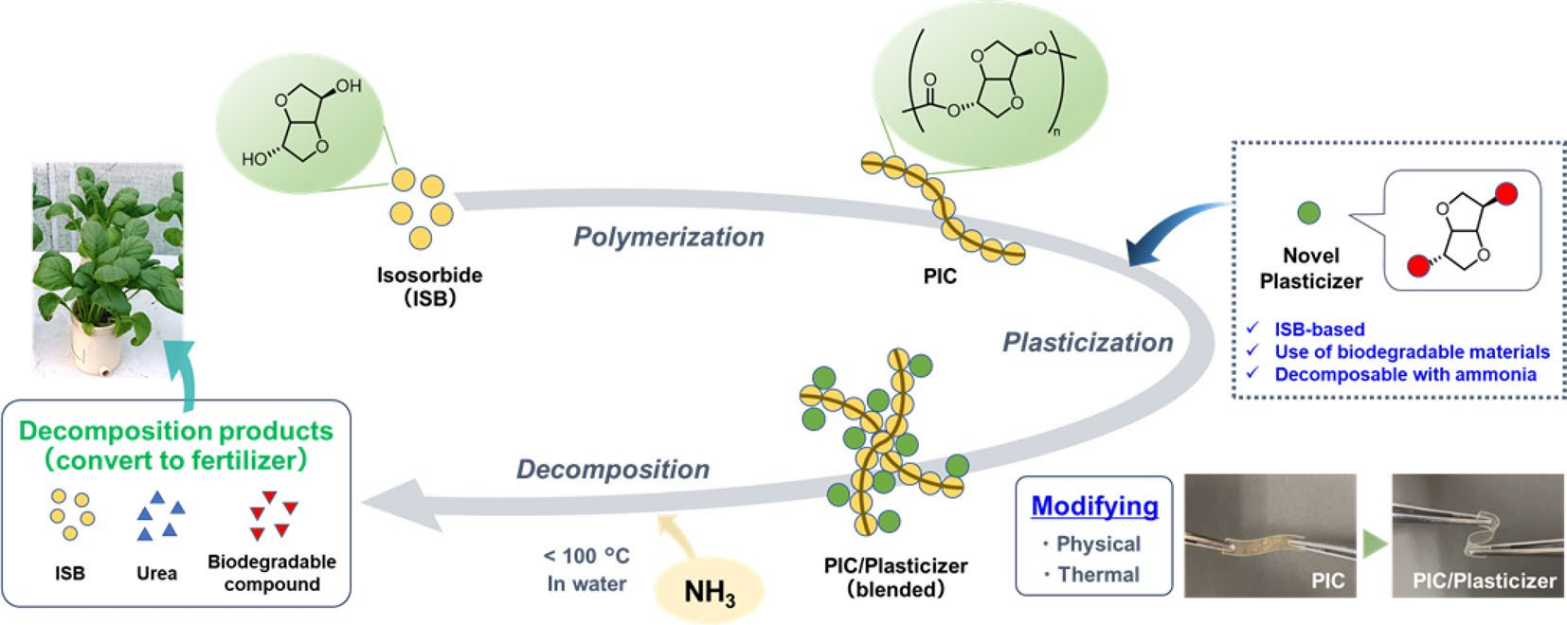
Schematic representation of the polymer system introduced in this work.

We hypothesized that ISB itself might be working as a fertilizer, and indeed, we demonstrated through plant experiments that ISB functions as a biostimulant that enhances plant growth.^30^ Building on our previous studies demonstrating that ISB-based polycarbonates can be converted into fertilizer-compatible components after use,^31–35^ the present study extends this concept through the development of dual-functional ISB-based plasticizers that simultaneously reduce the brittleness of PIC and retain compatibility with the previously established “plastics-to-fertilizer” ammonolysis system (Fig. 1). As PIC is inherently hard and brittle, we introduced novel dual functional ISB-based plasticizers that not only modify the physical properties of PIC but can also be decomposed by aqueous ammonia and subsequently utilized as fertilizers. Furthermore, the fertilizer effect of the resulting degradation products was demonstrated not only for model plants but also for edible plants.

Note that PIC is not a biodegradable polymer, and the material developed here is intended to function as a conventional plasticized polymer during use. The fertilizer function emerges only after end-of-life, when the whole polymer–plasticizer system is chemically transformed via ammonolysis into nutrient components.

### Synthesis of an ISB-based plasticizer

ISB, a bio-based compound and artificial product obtained from glucose, has been proven to function as a fertilizer, thus opening new possibilities for its use beyond that of a monomer. There have already been numerous reports on polymers based on ISB monomers, but considering that ISB can act as a fertilizer after having been used in a polymer, it is preferable to be used in polycarbonates because these can be decomposed upon treating with ammonia. As PIC itself is hard and brittle, copolymerization is the only way to adjust the physical properties of ISB-based polycarbonates.^36–38^ Aiming to modify the physical properties of PIC, novel plasticizers which can be decomposed by aqueous ammonia and used as a fertilizer in a manner similar to PIC are reported here.

**Scheme 1:**
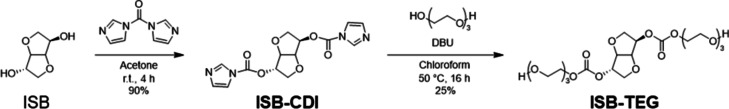
Synthesis of ISB-TEG.

To expand the concept of “plastics to fertilizer”, a new plasticizer for PIC, **ISB-TEG**, was designed based on a prediction made using Hansen solubility parameters (HSP). (Tables S1 and S2 as well as Figure S9). The good compatibility between **ISB-TEG** and PIC was supported by HSP analysis, giving a relative energy difference (RED) value of 0.87 (< 1) for the **PIC/ISB-TEG** pair. **ISB-TEG** was synthesized using the two-step process shown in **Scheme 1**. The ISB unit in **ISB-TEG**, which is the basic backbone of PIC, was sandwiched between triethylene glycol (TEG) units through carbonate linkages to endow it with the compatibility, flexibility, and processability required to improve the properties of hard and brittle PIC. Additionally, a noteworthy feature of **ISB-TEG** is its degradability. On account of the carbonate bonds in its structure, **ISB-TEG** was designed to decompose into ISB and TEG upon treatment with aqueous ammonia, in a manner similar to that of PIC. We hypothesized that a blend of PIC as the matrix polymer and **ISB-TEG**, abbreviated as **PIC/ISB-TEG**, would decompose after having been used as a plastic material upon treatment with aqueous ammonia, and to be directly used as a fertilizer given that ISB, TEG, and urea are all biodegradable.

The formation of reactive ISB, i.e., an imidazole intermediate of ISB (**ISB-CDI**), following treatment of ISB with carbonyldiimidazole was confirmed by means of ^1^H NMR spectroscopy (Figure S1). I**SB-TEG** was synthesized via a nucleophilic substitution reaction between **ISB-CDI** and excess TEG (**Scheme 1**). The successful synthesis of **ISB-TEG** was unequivocally confirmed using NMR spectroscopy and mass spectrometry (Figure S2).

### Effect of an ISB-based plasticizer on the properties of PIC

**Fig. 2:**
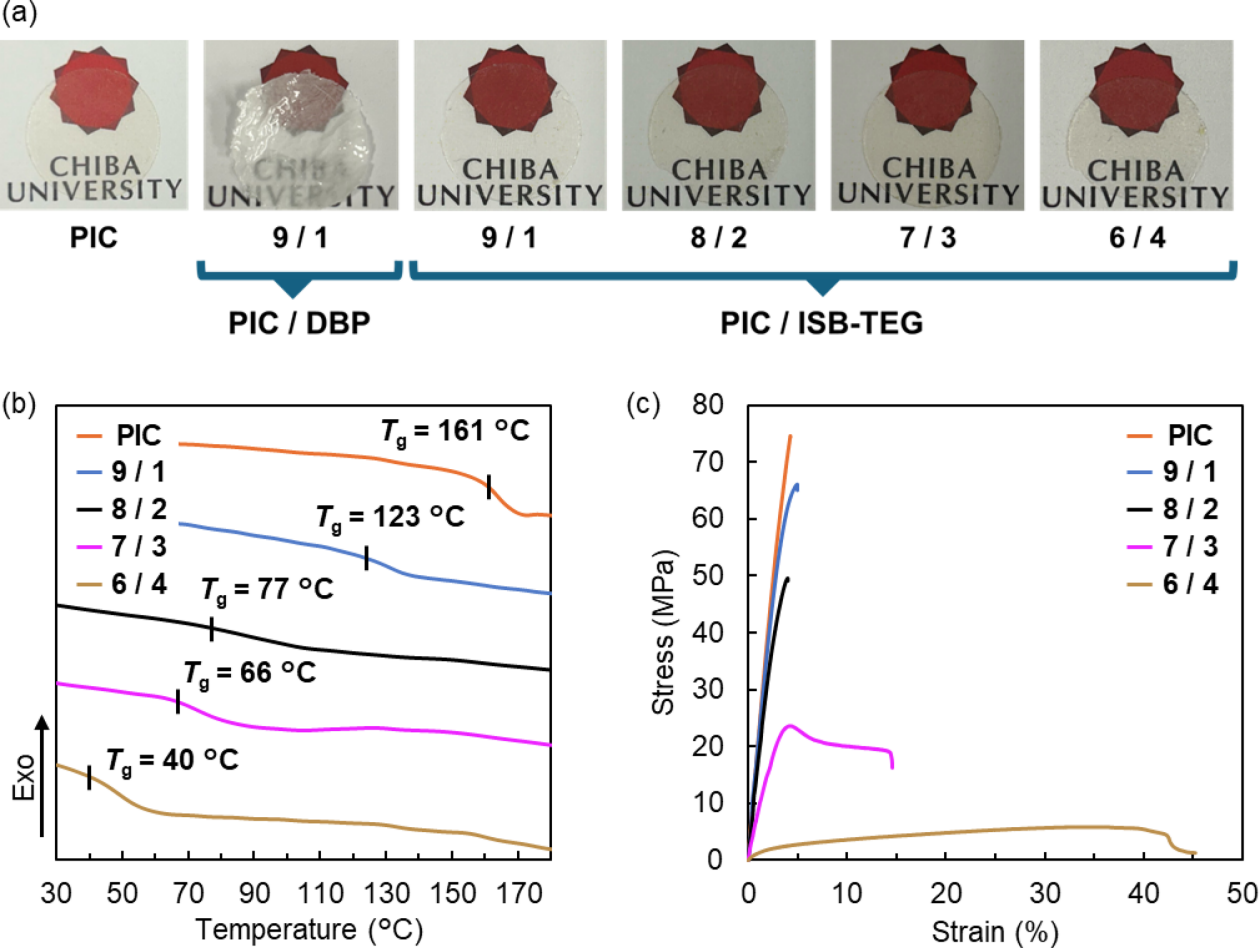
(**a**) Photographs of PIC, **PIC/DBP**, and **PIC/ISB-TEG** films. (**b**) DSC curves of **PIC/ISB-TEG** samples of various ratios (*T*_g_ values are given for each polymer; 2nd heating; 10 °C /min) (**c**) Stress–strain curves for PIC and **PIC/ISB-TEG** samples of various ratios.

For plasticizers, a high plasticizing efficiency and good compatibility with the main polymer are essential.^39^ As a reference, the commercially available and commonly used plasticizer dibutyl phthalate (DBP)^40,41^ was blended with PIC, which provided an opaque sample (**PIC/DBP** in Fig. 2a). This indicates that the blend of PIC and DBP causes phase separation and that the blend exists in a heterogeneous state. In contrast, when **ISB-TEG** was blended with PIC, the resulting blends **PIC/ISB-TEG** have good compatibility regardless of the mixing ratio. In short, PIC and **ISB-TEG** mix uniformly, as shown in Fig. 2a. The rational design of plasticizers based on HSP analysis resulted in excellent compatibility with PIC.

As effective plasticization lowers the polymer blend glass-transition temperature (*T*_g_) below that of the original polymer,^42,43^ enabling flexible rubber-like behavior. Hence, the *T*_g_ of **PIC/ISB-TEG**, a series of blended films of PIC, and **ISB-TEG** was measured using differential scanning calorimetry (DSC). As shown in Fig. 2b; Table 1, the *T*_g_ of neat PIC (161 °C) was not observed in the **PIC/ISB-TEG** blends and a new single *T*_g_, which decreased with increasing amount of **ISB-TEG**, was observed. This observation demonstrates that the compatibility between PIC and **ISB-TEG** is good. The *T*_g_ of **PIC/ISB-TEG** (w/w = 6/4) was even observed at 40 °C.

A plasticizer usually affects the mechanical properties of a polymer through decreasing tensile stress and increasing elongation at break relative to the neat polymer. The results of the tensile-stress and elongation-at-break tests conducted on **PIC/ISB-TEG** are summarized in Fig. 2c; Table 1. For neat PIC, the maximum stress was 74.6 MPa and the maximum strain was 4.3%. With increasing amount of plasticizer, the maximum stress decreased and the elongation at break increased, reflecting a significant change in physical properties (**Movie S1**). Combined, the results of the DSC measurements and the tensile tests show that **ISB-TEG** is as an effective plasticizer for PIC. The properties of PIC can be effectively tuned by using the **ISB-TEG** plasticizer. Moreover, by further varying the proportion of **ISB-TEG** in the blend, it can be expected that a library of polymers with a wide range of physical properties can be constructed.

This plasticization strategy induced larger changes in thermal properties, particularly *T*_g_, and mechanical properties than those reported for previously described isosorbide-based copolymer systems^28,29^. Specifically, in our previous studies, approximately 20 mol% of flexible comonomer units were introduced into isosorbide-based polymers. Although the incorporation of these flexible structures enabled *T*_g_ to be tuned by as much as approximately 60 °C, the flexibility could not be significantly improved, and the resulting materials remained hard and rigid, similar to the PIC homopolymer. In contrast, in the plasticizer-based system presented here, the addition of a similar amount of plasticizer enabled *T*_g_ to be changed by more than 80 °C, and even larger changes in *T*_g_ could be achieved at higher plasticizer contents. In this context, the plasticization strategy presented here represents a relatively simple and efficient approach for tuning the properties of PIC-based materials. Furthermore, unlike permanent copolymerization or polymer modification, the present approach preserves compatibility with the “plastics-to-fertilizer” degradation concept, in which the materials can be treated with aqueous ammonia after use and directly converted into fertilizers, as described below.

**Table 1:** DSC and tensile-test results for PIC and **PIC/ISB-TEG** samples of various ratios.

Entry	PIC/ISB-TEG (w/w)	T_g_^a^ (°C)	Young’s modulus^b^ (MPa)	Breaking stress^b^ (Mpa)	Breaking elongation^b^ (%)
1	10/0 (PIC)	161	2050	74.6	4.3
2	9/1	123	1870	65.9	5.0
3	8/2	77	1770	49.5	4.0
4	7/3	66	1020	23.5	14.6
5	6/4	40	140	5.9	45.2

^a^Measured by DSC (2nd heating; 10 °C /min).

^b^Estimated by stress−strain curves; experiments were conducted 3 times.

### Ammonolysis of PIC/ISB-TEG

**Scheme 2:**
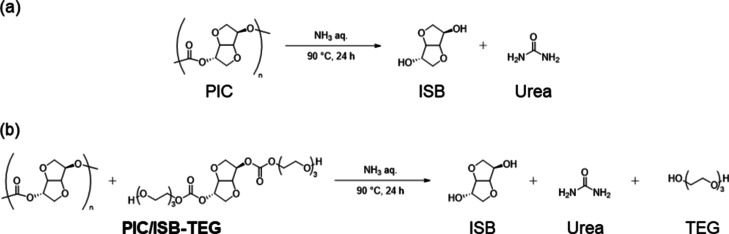
(**a**) Ammonolysis of PIC. (**b**) Ammonolysis of **PIC/ISB-TEG**.

**Fig. 3:**
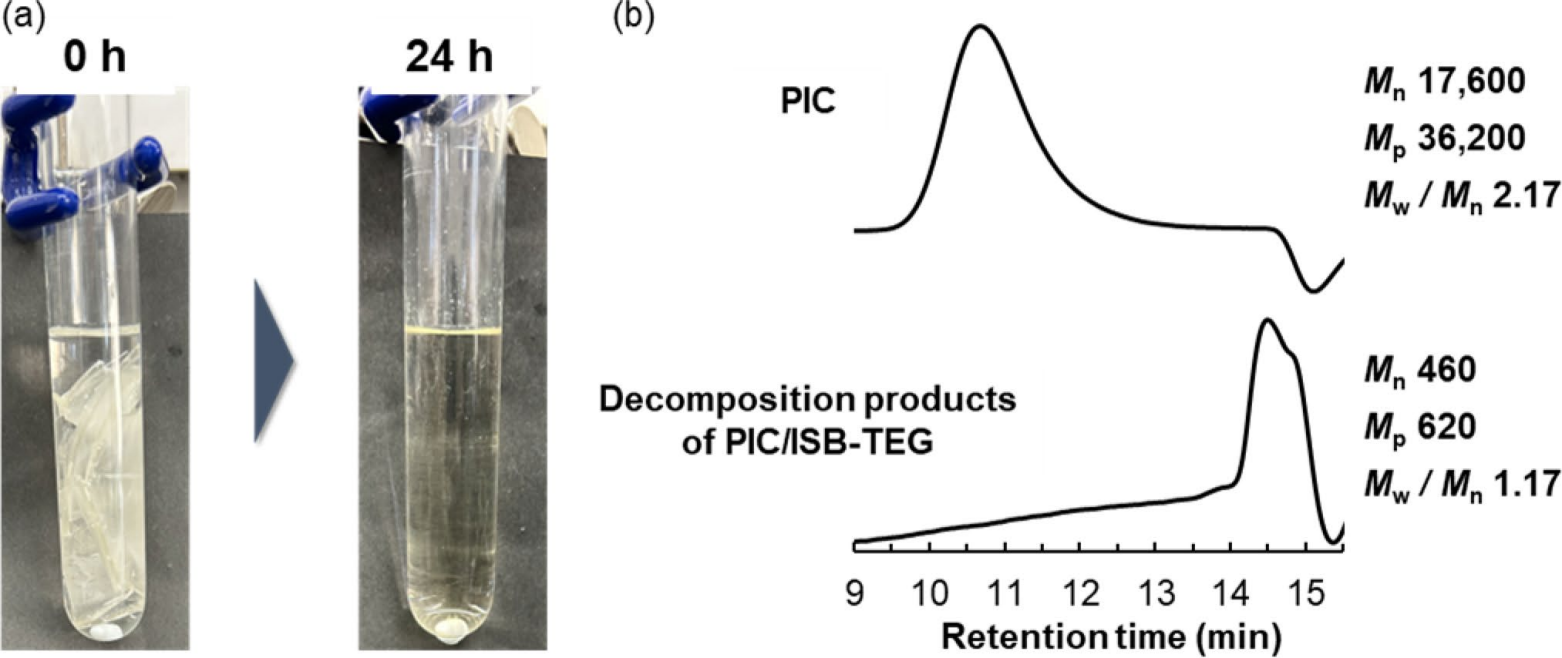
(**a**) Comparison of **PIC/ISB-TEG** before and after ammonolysis. (**b**) GPC profiles of PIC and the decomposition products (eluent: DMF; detector: RI; standards: polystyrene).

PIC undergoes ammonolysis to yield ISB and urea as a decomposition product (**Scheme 2a**). Building on this concept, the molecular design of **ISB-TEG** aims to achieve the conversion of waste plastic into fertilizer directly after use by decomposing **PIC/ISB-TEG** with ammonia. The decomposition products of **PIC/ISB-TEG** with ammonia are expected to be a mixture of urea, ISB, and TEG (**Scheme 2b**). We examined whether **PIC/ISB-TEG** could be decomposed by ammonia. Ammonolysis was conducted under the same conditions as the PIC decomposition reaction, i.e., 90 °C for 24 h (for details, see the ESI). The changes in appearance of **PIC/ISB-TEG** before and after ammonolysis are shown in Fig. 3a. The solid sample that existed before the reaction became a homogeneous aqueous solution after the reaction, suggesting that the decomposition proceeded well because PIC is insoluble in water while ISB, urea, and TEG are all soluble in water. Gel-permeation chromatography (GPC) profiles before and after ammonolysis show a big decrease in molecular weight (*M*_n_ = 17600 → < 500), thus corroborating the success of the degradation reaction (Fig. 3b). The decomposition products were also quantitatively evaluated using^1^ H NMR spectroscopy (Figure S3). The peaks observed for the decomposition products were identical to those of urea, ISB, and TEG, as the molecular design of **ISB-TEG** had intended. In this ammonolysis, the mass balance was calculated to be > 100%, which was attributed to the presence of additional water derived from the high hygroscopicity of TEG. The water content of the decomposition products was 10.1% of the total weight, as estimated by Karl-Fischer titration. The recovery rates derived from the^1^ H NMR analysis were 98.4% for ISB, 78.1% for urea, and 90.2% for TEG. The yield of urea was modest (78.1%), suggesting that partial decarboxylation of carbonate bonds occurred due to hydrolysis, thereby partially converting carbonate groups into carbon dioxide. This side reaction inevitably occurs to some extent under the present conditions.^26^ Further optimization of the ammonolysis conditions, including maintaining a sufficiently high ammonia concentration throughout the reaction to suppress competing hydrolysis, may improve the urea yield.

### Utilization of the degradation products as fertilizer

**Fig. 4:**
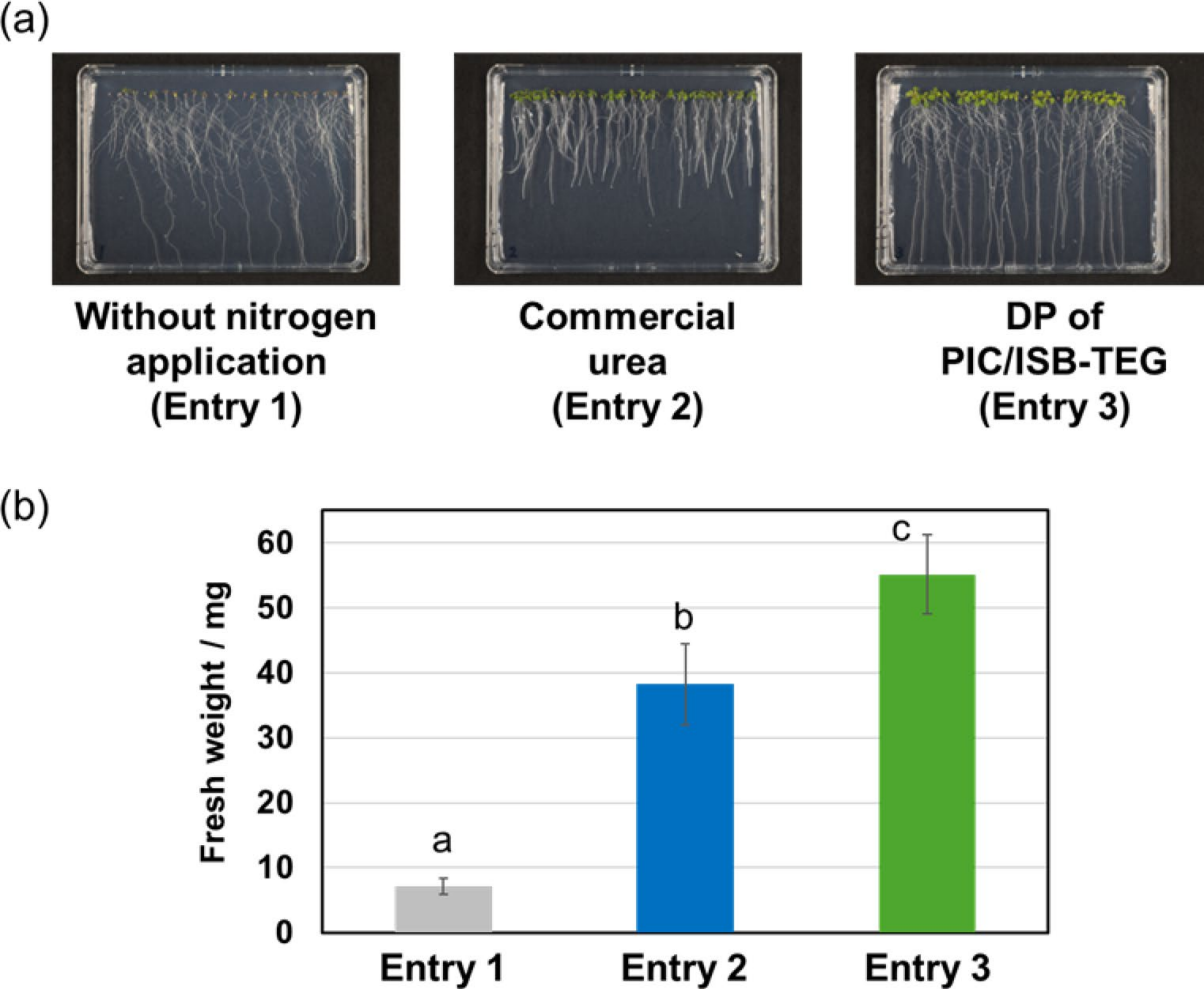
(**a**) Photographs and (**b**) fresh-weight values (10 plants) of *Arabidopsis thaliana* grown under three different conditions; values represent mean values of six replicates; error bars indicate the SD; mean values with the same letters are not significantly different as determined by a Tukey’s HSD test (*P* < 0.05); DP: degradation products.

To demonstrate a “plastics to fertilizer” approach with our mixed polymer and plasticizer system, a plant-growth test was conducted using the degradation products obtained from the ammonolysis of **PIC/ISB-TEG**. *Arabidopsis thaliana*, which was used as the model plant, was grown for 2 weeks (for details, see the ESI). Three aqueous solutions, i.e., a solution of commercially available urea (Fig. 4, entry 2), a solution of the **PIC/ISB-TEG**-degradation products (Fig. 4, entry 3) as well as a control sample without a fertilizer (Fig. 4, entry 1), were prepared as shown in Tables S3 and S4 and applied in the plant-growth test using *Arabidopsis thaliana*. The fresh-weight values and photographs of *Arabidopsis thaliana* after 2 weeks using the different solutions are presented in Fig. 4. *Arabidopsis thaliana* grew well in the presence of both the degradation products obtained from **PIC/ISB-TEG** (Fig. 4, entry 3) and urea (Fig. 4, entry 2), demonstrating that the degradation products from **PIC/ISB-TEG** did not have any negative impact on plant growth. *Arabidopsis thaliana* grown in the presence of the degradation products obtained from **PIC/ISB-TEG** (Fig. 4, entry 3) exhibited more growth than the control experiment with only urea (Fig. 4, entry 2), indicating that the combination of ISB with urea has a positive impact on plant growth. Although TEG is also present in the decomposition products, it constitutes only ca. 15% of the total weight of the decomposition products and is not considered to adversely affect the fertilization effect of urea and ISB.

The degradation products showed comparable or enhanced plant-growth behavior relative to the urea controls in some experiments; however, the present experimental design does not allow the individual contributions of ISB, TEG, and urea to be independently distinguished. Therefore, possible synergistic or biostimulant effects should be interpreted cautiously.

### Generality of the present “plastics to fertilizer” polymer system

**Fig. 5:**
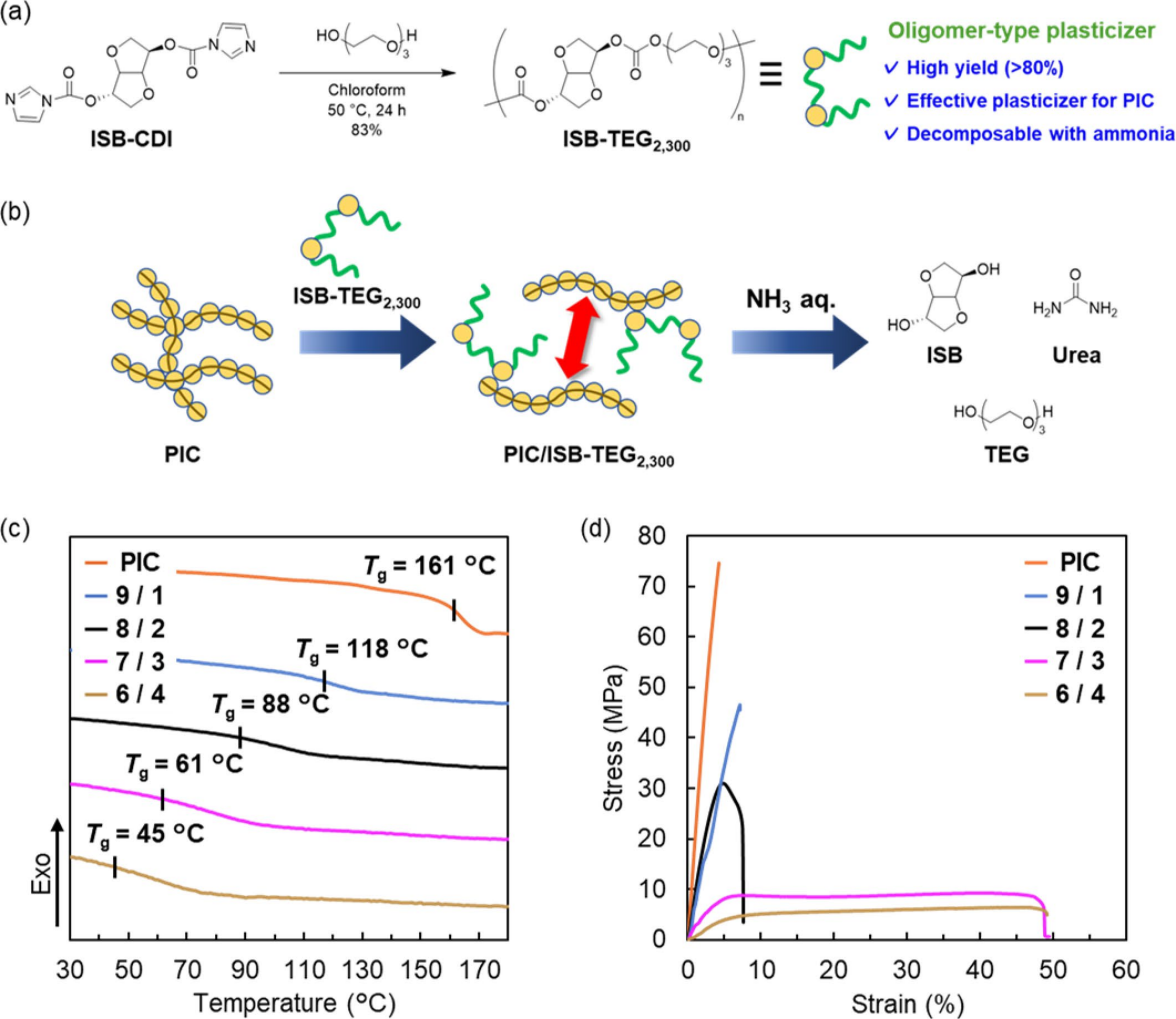
(**a**) Synthesis of **ISB-TEG**_**2,300**_. (**b**) Schematic representation of the effect of oligomer-type plasticizers on PIC. (**c**) DSC curves of **PIC/ISB-TEG**_**2,300**_ samples of various ratios (*T*_g_ values are given for each polymer; 2nd heating; 10 °C /min) (d) Stress–strain curves of PIC and **PIC/ISB-TEG**_**2,300**_ samples of various ratios.

Cultivation experiments with the model plant *Arabidopsis thaliana* confirmed that the degradation products can be used as effective fertilizers. The next step was to demonstrate this effect with edible crops, which requires larger-scale quantities of the degradation products. Another important consideration is that various ISB-based polymers have already been synthesized and are even commercially available, highlighting the need for a more convenient and efficient plasticizer synthesis to make this system broadly applicable. Since there are many examples of oligomer-type plasticizers being used,^44–47^ an oligomer-type plasticizer based on an ISB skeleton (**ISB-TEG**_**2,300**_) was synthesized (Fig. 5a). **ISB-CDI** and TEG were used as the monomers for the one-pot polycondensation, resulting in the successful synthesis of oligomer-type plasticizers with > 80% isolated yield (for details, see the ESI) and in gram-scale quantities sufficient for the minimum requirement to cultivate edible vegetables. We found that the **ISB-TEG**_**2,300**_ also works effectively as a plasticizer to modify thermal and physical properties of PIC as **ISB-TEG** (Fig. 5b, c, d, **Movie S2**). Although the decrease in *T*_g_ was comparable between **ISB-TEG**_**2,300**_ and **ISB-TEG** at the same additive content, the stress-strain curves revealed that **ISB-TEG**_**2,300**_ tended to enhance the elongation at break more significantly (Fig. 5c and d). Furthermore, another oligomer-type plasticizer (**ISB-DEG**_**2,300**_), synthesized by using diethylene glycol (DEG) as a monomer, also worked effectively as a plasticizer for PIC (Figure S8, Movie S2).

Although ISB-TEG was obtained in a relatively low isolated yield (less than 25%), it demonstrated the feasibility of a “plastics-to-fertilizer” concept that includes plasticizer-derived components. In contrast, the oligomer-type plasticizers were synthesized in substantially higher yields (over 80%) and are considered more promising from the viewpoints of synthetic practicality and scalability.

### Utilization of the degradation products from the plastic system as fertilizers for the growth of edible vegetables

**Fig. 6:**
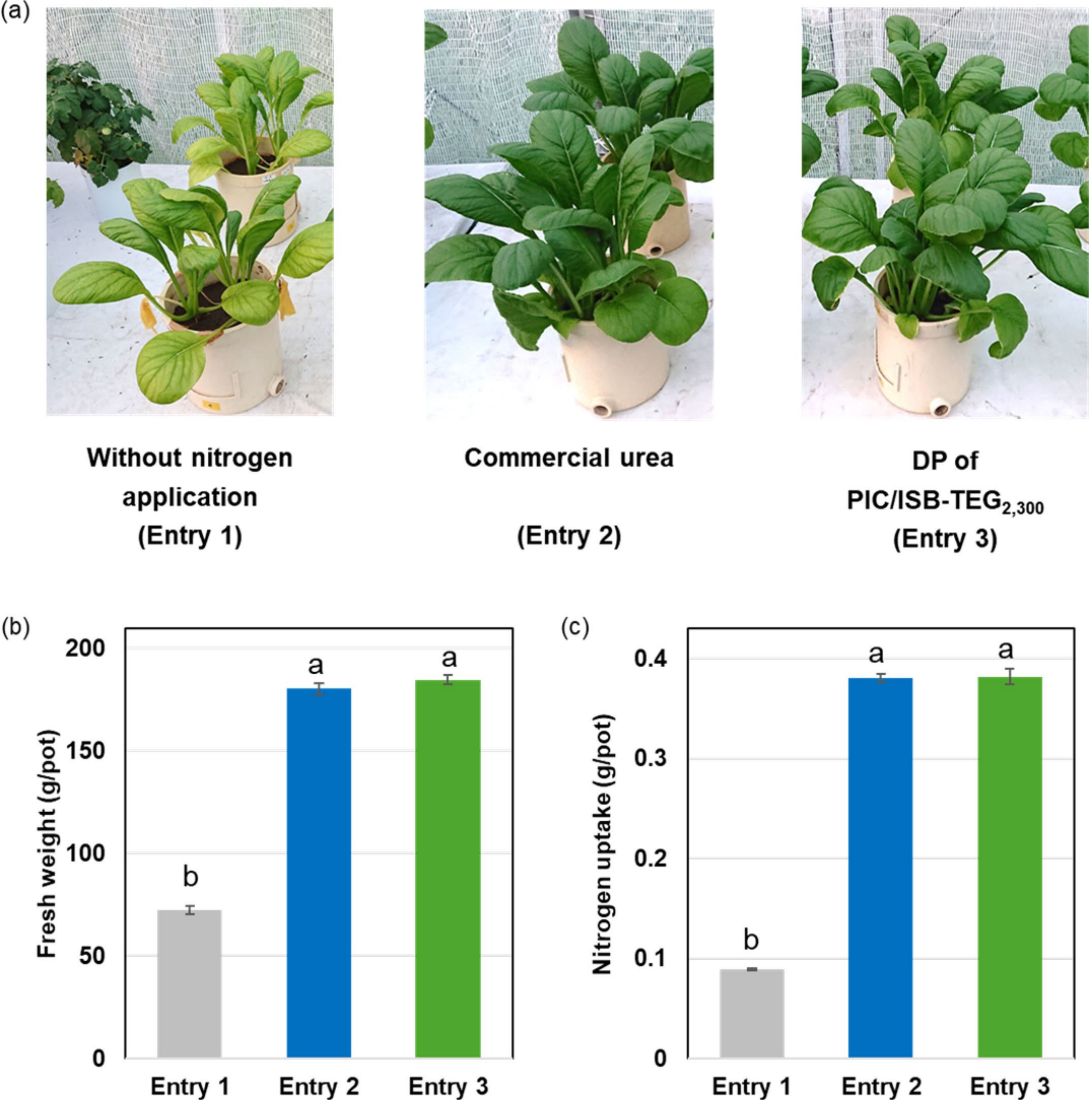
(**a**) Photographs of komatsuna (*Brassica rapa* var. *perviridis*) grown in soil treated with the degradation products of **PIC/ISB-TEG**_**2,300**_ and reference fertilizers, i.e., no nitrogen source applied (entry 1), commercial urea (entry 2), and the degradation products of **PIC/ISB-TEG**_**2,300**_ (entry 3). The photographs were taken one day before harvesting; DP: degradation products. (**b**) Fresh-weight values of komatsuna (*Brassica rapa* var. *perviridis*) grown in soil and treated with entries 1–3; values represent the mean values of five replicates; error bars indicate the SE; mean values with the same letters are not significantly different as determined by a Tukey’s HSD test (*P* < 0.05). (**c**) Nitrogen uptake of komatsuna (*Brassica rapa* var. *perviridis*) grown in soil and treated with entries 1–3; values represent the mean values of five replicates; error bars indicate the SE; mean values with the same letters are not significantly different as determined by a Tukey’s HSD test (*P* < 0.05).

Experiments using *Arabidopsis thaliana* demonstrated that ISB can be used as a fertilizer; here we show that the decomposition products of the ISB-based polymers with plasticizer obtained from ammonolysis can be used to grow the edible vegetable komatsuna (*Brassica rapa* var. *perviridis*) in soil (Fig. 6a). A pot-cultivation test for komatsuna plants was conducted using the degradation products obtained from the ammonolysis of (1) **PIC/ISB-TEG**_**2,300**_ and (2) commercially available urea. A cultivation without the application of a nitrogen source (no treatment with a nitrogen-based fertilizer) was also conducted in order to clarify if the ammonolysis decomposition products of the ISB-based polymers with plasticizer are effective nitrogen-based fertilizers. The amount of nitrogen applied from the degradation products of **PIC/ISB-TEG**_**2,300**_ and that of commercial urea was identical, i.e., 0.50 g of nitrogen per 0.02 m^2^ Wagner pot. Phosphorus and potassium fertilizers were added as P_2_O_5_ (0.5 g/pot) and K_2_O (0.5 g/pot) to all cultivations. The komatsuna plants were grown for 36 days in a greenhouse with daily irrigation to 60% of the maximum water holding capacity of the soil. The degradation products of **PIC/ISB-TEG**_**2,300**_ were effective as a nitrogen-based fertilizer for komatsuna. Compared to the treatment without a nitrogen source, the treatment of the degradation products of **PIC/ISB-TEG**_**2,300**_ resulted in significantly higher yields, as measured by the fresh weight, and was comparable to that of a commercial urea treatment (Fig. 6b). The nitrogen uptake by the komatsuna was also significantly higher during the treatment with the degradation products obtained from **PIC/ISB-TEG**_**2,300**_ compared to the treatment without a nitrogen source (Fig. 6c). The results of the fresh-weight and nitrogen-uptake analyses indicate that the use of the degradation products of **PIC/ISB-TEG**_**2,300**_ as a nitrogen-based fertilizer is as effective as using commercial urea. The different growth responses observed between *Arabidopsis thaliana* and komatsuna may be attributable to species-dependent effects. In our previous study using ammonolysis products derived from PIC itself^48^, cultivation of komatsuna showed plant-growth behavior comparable to that of the urea control, whereas the enhanced growth effect observed relative to urea in *Arabidopsis thaliana* was not observed. These results suggest that the apparent fertilization or biostimulant effects of the degradation products may depend on the plant species and cultivation conditions.

## Conclusions

In this study, building on our previous findings that isosorbide-based polycarbonates can be converted into fertilizer-compatible components through ammonolysis after use, we developed a plasticizer-containing PIC system employing dual-functional isosorbide-based plasticizers. These plasticizers simultaneously tune the physical properties of PIC while remaining convertible into fertilizer-compatible components after use, thereby extending the previously established “plastics-to-fertilizer” concept through additive-based polymer design.

Our findings demonstrate that plasticizer-containing plastics can be transformed from waste into valuable products that function as fertilizers and support plant growth. In this study, the PIC polymer was employed as a model system to demonstrate the feasibility of this concept; however, the molecular weight and mechanical properties of the present materials are still insufficient for practical applications requiring high mechanical performance. Nevertheless, further improvement of the material properties should be possible through the use of PIC-related polymers with intrinsically robust mechanical properties.

Furthermore, the present plasticization strategy may enable the development of flexible plastic materials, such as films, plastic bags, and other soft plastic products where flexibility is required. The possibility of transforming such soft plastic materials into fertilizers after use through simple ammonolysis treatment represents an attractive direction for future sustainable plastics.

This study also represents the first demonstration of cultivating edible vegetables using fertilizer derived from a plasticizer-containing PIC system, whereas our previous studies only demonstrated this concept using degradation products derived from PIC itself without plasticizer components^48^.

Although good compatibility between PIC and the ISB-based plasticizers was observed in freshly prepared materials, evaluation of the practical applicability of the ISB-based plasticizer systems, including long-term blend stability and plasticizer migration behavior, remains an important subject for future studies.

Although the overall environmental and energy balance of the process has not yet been fully evaluated, further assessment of process sustainability, including energy consumption and aqueous product handling, will be important in future studies. Nevertheless, by integrating polymer materials with the nitrogen cycle, our approach may provide a useful framework for designing plastics with more sustainable end-of-life pathways.

## Supplementary Information

Below is the link to the electronic supplementary material.

Supplementary Material 1

Supplementary Material 2

Supplementary Material 3

## Data Availability

The data that support the findings of this study are available from the corresponding author upon reasonable request.
