# Supplementary material for: Plastics to fertilizer: A polymer system based on isosorbide as a monomer, plasticizer, and fertilizer
Source: Sci Rep. 2026 Aug 18;16:23701. doi: 10.1038/s41598-026-63638-1 (PMC13487305; doi:10.1038/s41598-026-63638-1)
Supplement: Supplementary file 1 [file 41598_2026_63638_MOESM3_ESM.pdf]

Electronic supplementary information

Plastic to fertilizer: a polymer system based on isosorbide as a monomer, plasticizer, and fertilizer

Shunsuke Fujimata,[1] Yoshiki Tokonami,[2] Raj Kishan Agrahari,[3] Toyokazu Tsutsuba,[1] Kazuaki Rikiyama,[1] Norio Tomotsu,[1] Tatsuo Taniguchi,[1] Takehiro Kamiya*[3], Mizuhiko Nishida*[2] and Daisuke Aoki*[1,4]

[1]Department of Applied Chemistry and Biotechnology, Graduate School of Engineering, Chiba University, 1-33 Yayoi-cho, Inage-ku, Chiba-shi, Chiba 263-8522, Japan

[2]Field Science Center, Graduate school of agricultural science, Tohoku University, 232-3, Yomogida, Naruko Onsen, Osaki, Miyagi, 989-6711, Japan

[3]The Laboratory of Plant Nutrition and Fertilizers, Graduate School of Agricultural and Life Sciences, The University of Tokyo, 1-1-1, Yayoi, Bunkyo-ku, Tokyo 113-8657, Japan

[4]Research Center for Space Agriculture and Horticulture, Chiba University, 648 Matsudo, Matsudo-shi, Chiba 271-8510, Japan

Table of contents

1. Reagents and measurements ……………………………………………………………………….S2
2. Experimental procedure…………………………………………………………………………….S4
3. Figures and tables ……………………………………………………………………………….S8
4. Plant-growth test ……………………………………………………………………………….S16
5. References……………………………………………………………………………………….S18

1. Reagents and measurements

Reagents

All reagents and solvents were purchased from Tokyo Chemical Industry Co., Ltd. (Tokyo, Japan), Kanto Chemical Co., Inc. (Tokyo, Japan), FUJIFILM Wako Pure Chemical Corp. (Osaka, Japan), and Merck KGaA (Darmstadt, Germany), and were used without further purification. Poly(isosorbide carbonate) (PIC, M_n = 17,600) was synthesized according to a previously reported procedure[1].

Measurements

Spectroscopy

^{1}H NMR spectra of the products were recorded at 500 MHz on a Bruker AVANCE NEO 500 spectrometer in DMSO-d_6 and chloroform-*d* ($CDCl_3$) with tetramethylsilane as an internal standard.

Molecular weights

The molecular weights of the products were determined by JASCO HSS-1500 GPC system equipped with a guard column (TOSOH TSKgel® guard column SuperH-L), three columns (TOSOH TSKgel® SuperH 6000, 4000, and 2500), and a refractive index (RI) detector. *N,N*-Dimethylformamide (DMF) with lithium bromide (5.0 mM) was used as the eluent for GPC at a flow rate of 0.6 mL min^{-1} at 40 °C. Polystyrene standards (number average molecular weight (M_n) 1920–2630000 g mol^{-1}; polydispersity index (PDI) = 1.03–1.08) were used to calibrate the GPC system.

Thermal property

The glass transition temperatures (T_g) of PIC and plasticized PIC specimens were determined by differential scanning calorimetry (DSC) on a SHIMADZU DSC-60A Plus. Each sample was heated at a rate of 10 °C min^{-1} under a flow of nitrogen. The T_g values were determined from the 2nd heating scan.

Mechanical properties

The tensile tests of specimens were conducted using a Shimadzu Autograph AGS-X universal testing machine with TCE-N300-CE. The maximum load capacity of the machine was 100 N, and the crosshead displacement was controlled with a precision of ±0.5%. The tests were performed at room temperature under a tensile rate of 10 mm/min on three samples under the same conditions. The Young's modulus was defined as the ratio of tensile stress to tensile strain in the linear elastic region of the material. The breaking stress and breaking elongation were determined from the stress-strain curve as the maximum stress and the strain at break, respectively.

Preparation of solvent-cast films

The mixtures containing PIC and 10, 20, 30, and 40 wt% of plasticizer, with a total mass of 0.2 g, were dissolved in 5.0 mL of chloroform and stirred at room temperature for 12 h. The solution was poured into a glass Petri dish (φ75 mm) and placed on a hot plate at 50 °C overnight, covered with aluminum foil. The resulting films were carefully peeled and subsequently dried in a vacuum oven for 12 h to remove residual solvent.

Preparation of melt-pressed specimens

The dumbbell-shaped tensile specimens of PIC and plasticized PIC were prepared using a MeltPrep VCM (Vacuum Compression Molding) system. The cast films of the polymers were partly placed in a mold, melted at 100–215 °C for 10 min, followed by compression molding under vacuum and subsequent cooling to room temperature under the same vacuum

condition.

2. Experimental procedure

2.1. Synthesis of ISB-CDI[2]

Scheme S1. Synthesis of **ISB-CDI**.

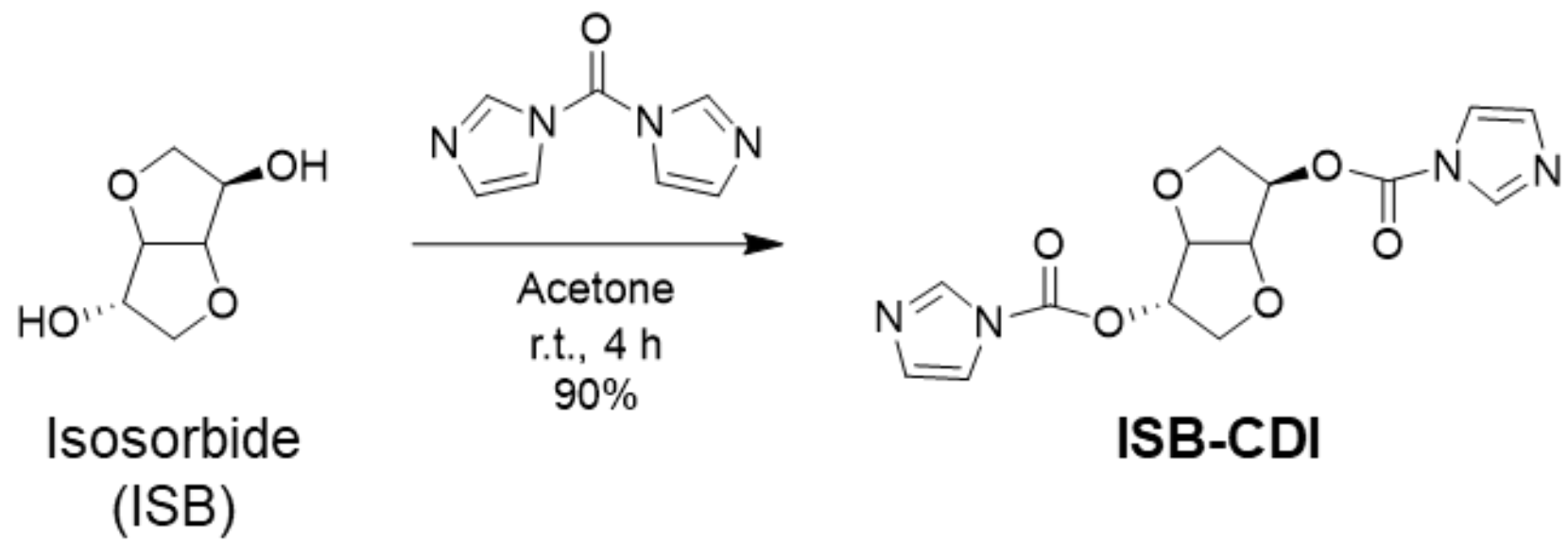

Isosorbide (ISB) (1.00 g, 6.84 mmol) was dissolved in acetone (20 mL). 1,1'-carbonyldiimicazole (CDI) (2.78 g, 17.1 mmol) was slowly added and the reaction mixture was stirred under nitrogen at room temperature for 4 h. The resulting precipitate was filtered and washed with diethyl ether to obtain **ISB-CDI** as a white powder (2.06 g, 90.0%).

^{1}H NMR (500 MHz, 298 K, $CDCl_3$): δ 8.15 (d, J = 27 Hz, 2H), 7.43 (d, J = 27 Hz, 2H), 7.11 (d, J = 11 Hz, 2H), 5.48 (d, J = 2.9 Hz, 1H), 5.41–5.45 (m, 1H), 5.10 (t, J = 5.3 Hz, 1H), 4.70 (d, J = 4.9 Hz, 1H), 4.12–4.21 (m, 2H), 4.02–4.07 (m, 2H).

2.2. Synthesis of ISB-TEG

Scheme S2. Synthesis of **ISB-TEG**.

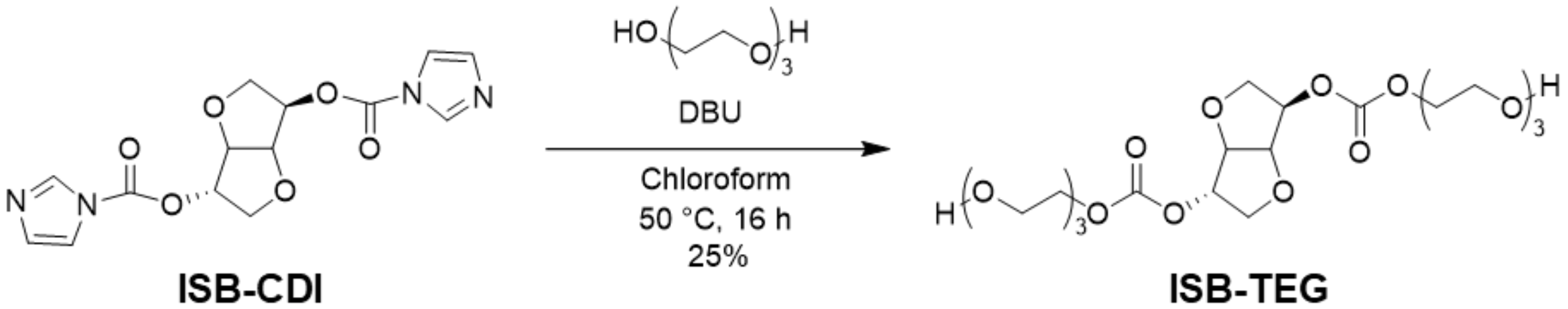

ISB-CDI (1.41 g, 4.18 mmol), triethylene glycol (TEG) (1.89 g, 12.6 mmol) and 1,8-diazabicyclo[5.4.0]undec-7-ene (DBU) (13.7 mg, 83.6 μmol) were dissolved in chloroform (17 mL). The reaction mixture was stirred at 50 °C for 16 h. The solution was washed with 1N hydrochloric acid and distilled water, dried over anhydrous Na_2SO_4, filtered, and evaporated. The crude product was purified by flash column chromatography (chloroform / methanol = 18/1) to obtain **ISB-TEG** as a colorless oil (0.51 g, 24.5%).

^{1}H NMR (500 MHz, 298 K, $CDCl_3$): δ 5.05–5.11 (m, 2H), 4.89 (t, J = 4.9 Hz, 1H), 4.56 (d, J = 4.5 Hz, 1H), 4.29–4.35 (m, 4H), 4.09 (d, J = 11 Hz, 1H), 4.00–4.04 (m, 1H), 3.87–3.94 (m, 2H), 3.71–3.76 (m, 8H), 3.65–3.69 (m, 8H), 3.61 (t, J = 4.7 Hz, 4H); ^{13}C NMR (125 MHz, 298 K, $CDCl_3$): δ 154.48, 154.18, 85.82, 81.29, 80.91, 73.21, 72.56, 70.66, 70.49, 70.33, 70.31, 68.82, 68.78, 67.32, 67.27, 61.74; ESI-MS (m/z): calcd for $[M + Na]^+$, $C_{20}H_{34}O_{14}Na$, 521.1841; found, 521.1828.

2.3. Ammonolysis of PIC/ISB-TEG

Scheme S3. Ammonolysis of **PIC/ISB-TEG**.

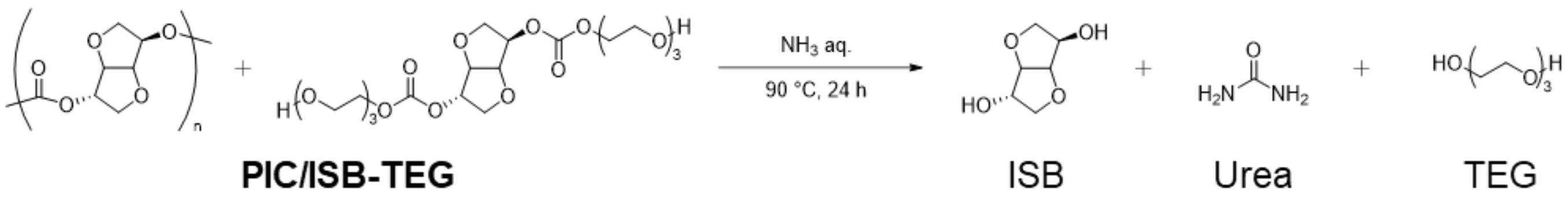

PIC/ISB-TEG (PIC 0.416 g, **ISB-TEG 0.177 g**) was placed in a 50 mL reactor. An aqueous ammonia solution was added to the reactor in an amount corresponding to 30 equivalents relative to the carbonate bonds in the polymer and plasticizer. The reaction mixture was heated at 90 °C for 24 h. Then, the resulting solution was evaporated to afford the decomposition products as a brown solid (0.857 g). The water content of the decomposition product, determined by Karl Fischer titration (MKC-710M, Kyoto Electronics Manufacturing Co., Ltd.), was 10.1 wt%. The yields of ISB, urea, and TEG were 0.398 g (98.4%), 0.147 g (78.1%), and 0.097 g (90.2%), respectively.

2.4. Synthesis of ISB-TEG$_{2,300}$

Scheme 4. Synthesis of **ISB-TEG$_{2,300}$**.

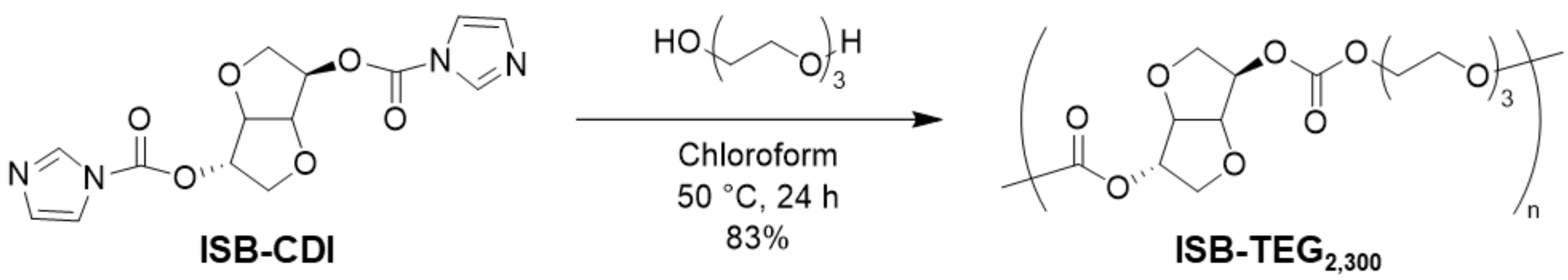

ISB-CDI (1.50 g, 4.49 mmol) and triethylene glycol (TEG) (1.01 g, 6.74 mmol) were dissolved in chloroform (11 mL). The reaction mixture was stirred under nitrogen atmosphere at 50 °C for 24 h. Upon completion of the reaction, the resulting solution was slowly added to a mixture of hexane/ethanol (6/4 = v/v, 100 mL) to precipitate the desired product. The product was collected by centrifugation, and the supernatant was decanted. The residue was dried under vacuum to obtain **ISB-TEG$_{2,300}$** as a colorless oil (1.30 g, 83.0%).

^{1}H NMR (500 MHz, 298 K, $CDCl_3$): δ 5.05–5.13 (m, 2H), 4.86–4.92 (m, 1H), 4.52–4.58 (m, 1H), 4.26–4.36 (m, 4H), 4.08 (d, J = 9.2 Hz, 1H), 4.01 (d, J = 10.8 Hz, 1H), 3.90 (d, J = 5.0 Hz, 2H), 3.69–3.76 (m, 5H), 3.63–3.69 (m, 5H), 3.60 (t, J = 5.0 Hz, 1H).

2.5. Synthesis of ISB-DEG$_{2,300}$

Scheme S5. Synthesis of **ISB-DEG$_{2,300}$**.

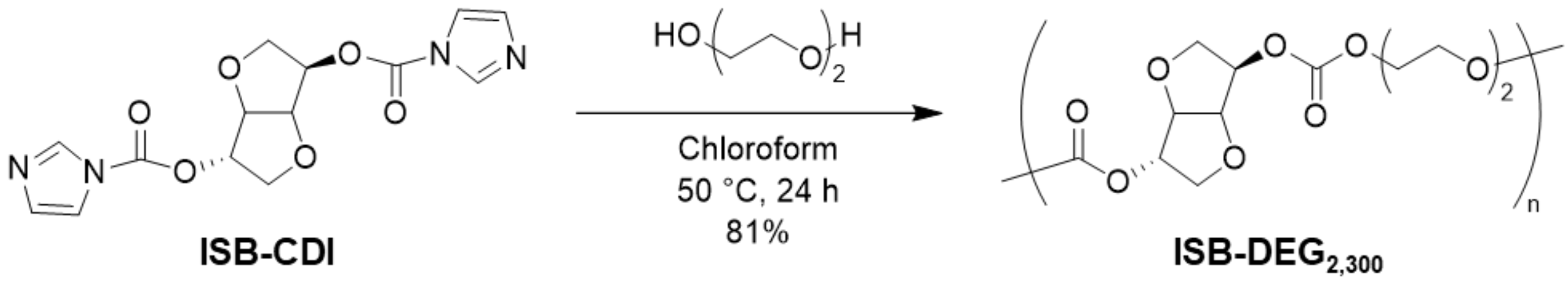

ISB-CDI (1.50 g, 4.49 mmol) and diethylene glycol (DEG) (0.72 g, 6.74 mmol) were dissolved in chloroform (10 mL). The reaction mixture was stirred under nitrogen atmosphere at 50 °C for 24 h. Upon completion of the reaction, the resulting solution was slowly added to a mixture of hexane/ethanol (6/4 = v/v, 100 mL) to precipitate the desired product. The product was collected by centrifugation, and the supernatant was decanted. The residue was dried under vacuum to obtain **ISB-DEG$_{2,300}$** as a colorless oil (1.10 g, 80.5%).

^{1}H NMR (500 MHz, 298 K, $CDCl_3$): δ 5.05–5.15 (m, 2H), 4.90 (t, J = 4.6 Hz, 1H), 4.55 (d, J = 4.3 Hz, 1H), 4.26–4.39 (m, 4H), 3.87–4.12 (m, 4H), 3.69–3.79 (m, 4H), 3.61 (t, J = 4.5 Hz, 1H).

2.6. Synthesis of PIC

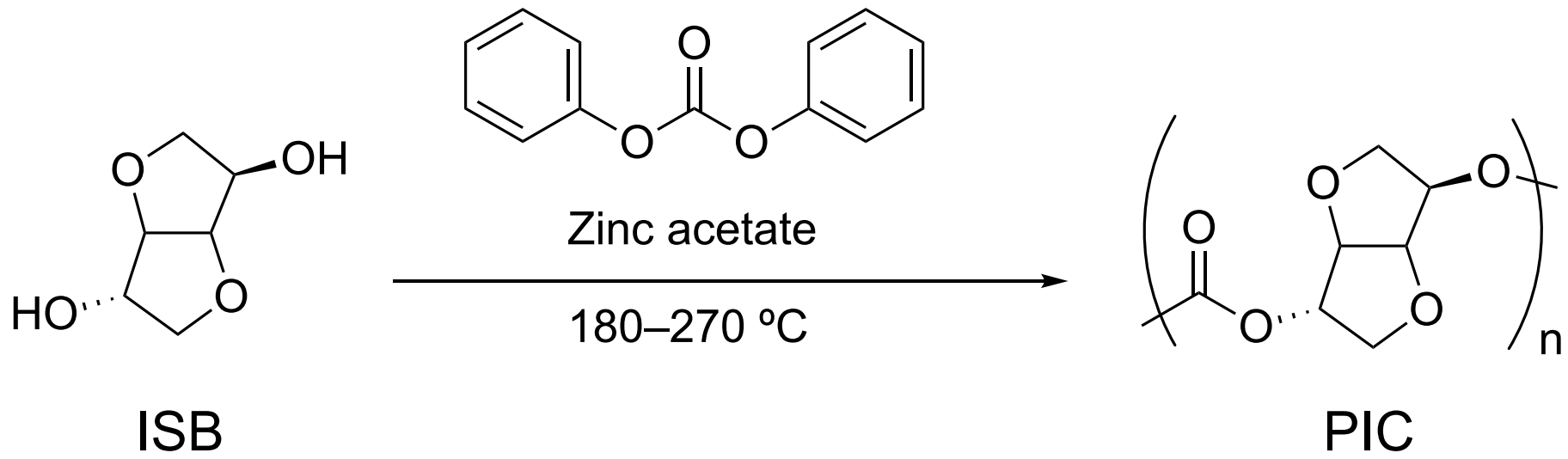

PIC was synthesized by a one-pot melt polycondensation method, in which transesterification and subsequent polycondensation were carried out sequentially in the same reactor. ISB (38.20 g, 0.261 mol), DPC (56.01 g, 0.261 mol), and zinc acetate (21.2 mg, 0.116 mmol, 0.05 mol% for ISB) were charged into a 200 mL three-necked round-bottomed flask equipped with a mechanical stirrer. In the transesterification stage, the reactants were heated to 180 °C under a nitrogen atmosphere and stirred for 30 min. The temperature was then gradually increased to 200 °C and maintained for an additional 30 min. Subsequently, the reaction entered the polycondensation stage, during which the temperature was increased to 220 °C and maintained for 30 min under reduced pressure (ca. 10 mmHg) to remove phenol. The temperature was then further increased to 240 °C and maintained for 1.5 h under high vacuum (<0.2 mmHg). Then, the temperature was further increased to 270 ºC and maintained for 1.5 h under high vacuum (<0.2 mmHg). The system was cooled to room temperature under a nitrogen atmosphere. The crude product was dissolved in chloroform and poured into methanol. The precipitate was collected and dried under vacuum to afford PIC (43.42 g, 96.6%, M_n = 17,600) as a white solid. ^{1}H NMR spectrum and GPC chart of PIC are shown in Figure S10.

2.7. Solubility test for ISB-TEG[3]

Hansen solubility parameters (HSPs) were obtained using the HSPiP software (Version 5.4.08 Hansen-Solubility.com), which implements Hansen's three-dimensional solubility parameter theory.

Theory of HSPiP

The Hansen solubility parameter is defined by the following equation:

$$\delta_t = \sqrt{\delta_D^2 + \delta_P^2 + \delta_H^2} \tag{a}$$

where δ_t, δ_D, δ_P and δ_H represent the total, dispersion, polar, and hydrogen-bonding solubility parameters, respectively.

To evaluate the difference in solubility between two substances, the solubility distance R_a is defined as:

$$R_a = \sqrt{4(\delta_{D1} - \delta_{D2})^2 + 4(\delta_{P1} - \delta_{P2})^2 + 4(\delta_{H1} - \delta_{H2})^2} \tag{b}$$

A smaller R_a value indicates that the intermolecular interactions of the two substances are similar, suggesting higher mutual miscibility. The concept of HSPs can be visualized by plotting δ_D, δ_P, and δ_H in a three-dimensional space, known as the Hansen solubility sphere. In this representation, good solvents are located inside the sphere, while poor solvents lie outside. The HSP of the target material is represented at the center of the sphere, and the radius of the sphere is defined as the interaction radius R_0.

Another useful parameter is the relative energy difference (RED), defined as:

$$RED = \frac{R_a}{R_0} \tag{c}$$

A value of $RED \leq 1.0$ indicates a good solvent, while higher values correspond to poorer solvents.

Using this theoretical framework, the HSP of PIC was determined. The novel plasticizer, **ISB-TEG**, exhibited a *RED* value below 1.0, indicating a high level of compatibility with PIC (**Table S2**, **Figure S9**).

3. Figures and tables

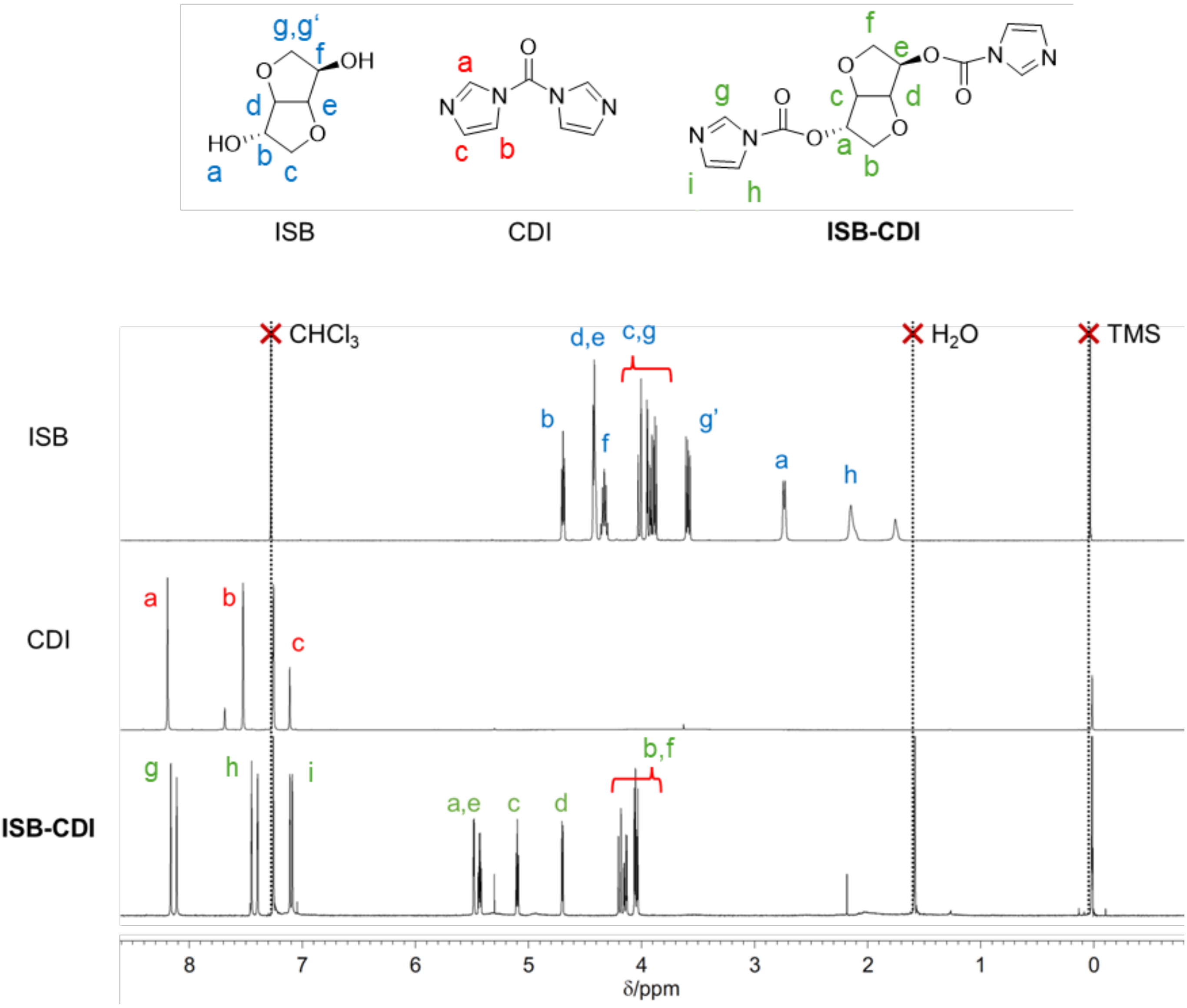

Figure S1. ^{1}H NMR spectra of ISB, CDI, and **ISB-CDI** (500 MHz, 298 K, $CDCl_3$).

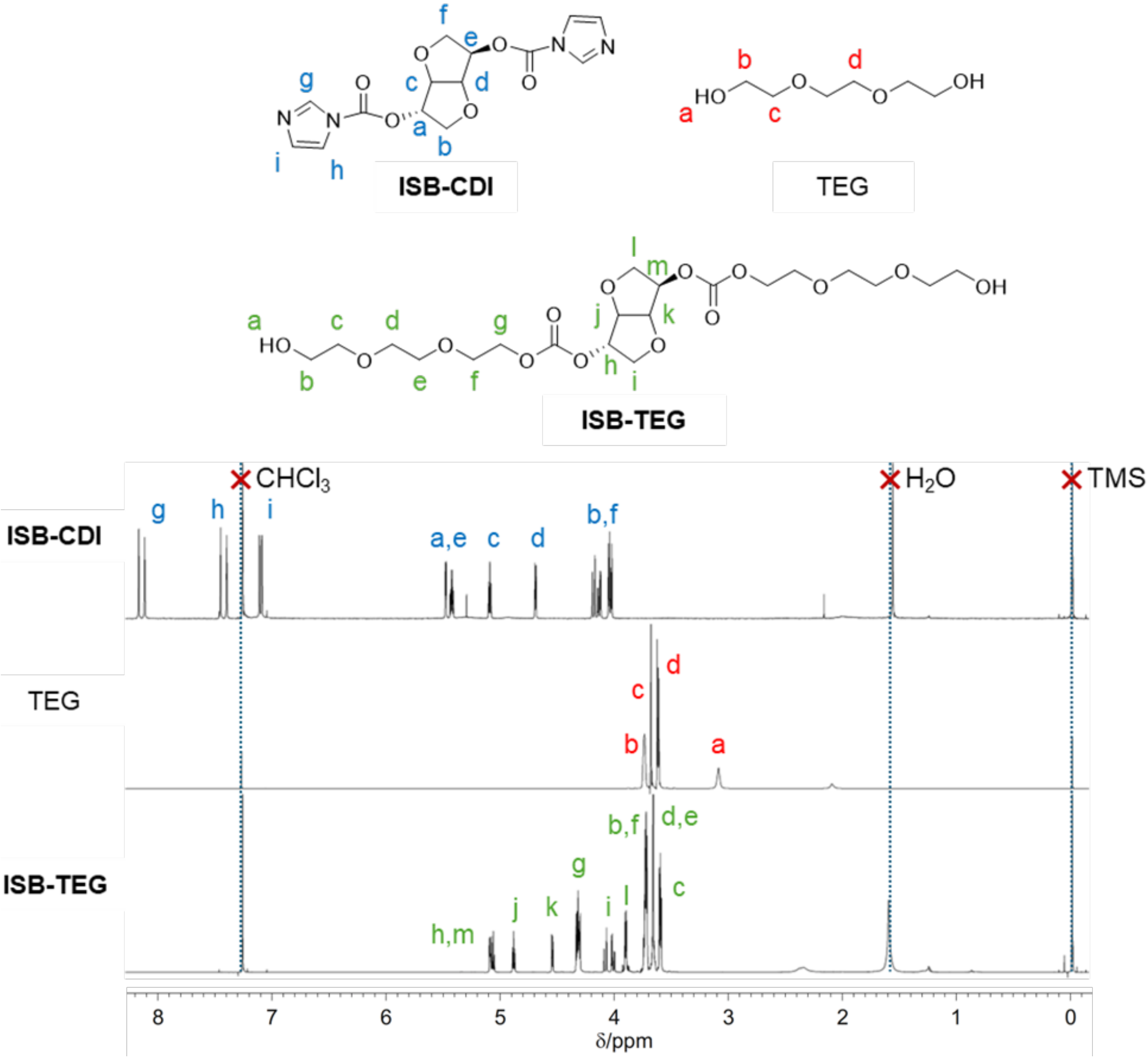

Figure S2. ^{1}H NMR spectra of **ISB-CDI,** TEG, and **ISB-TEG** (500 MHz, 298 K, $CDCl_3$).

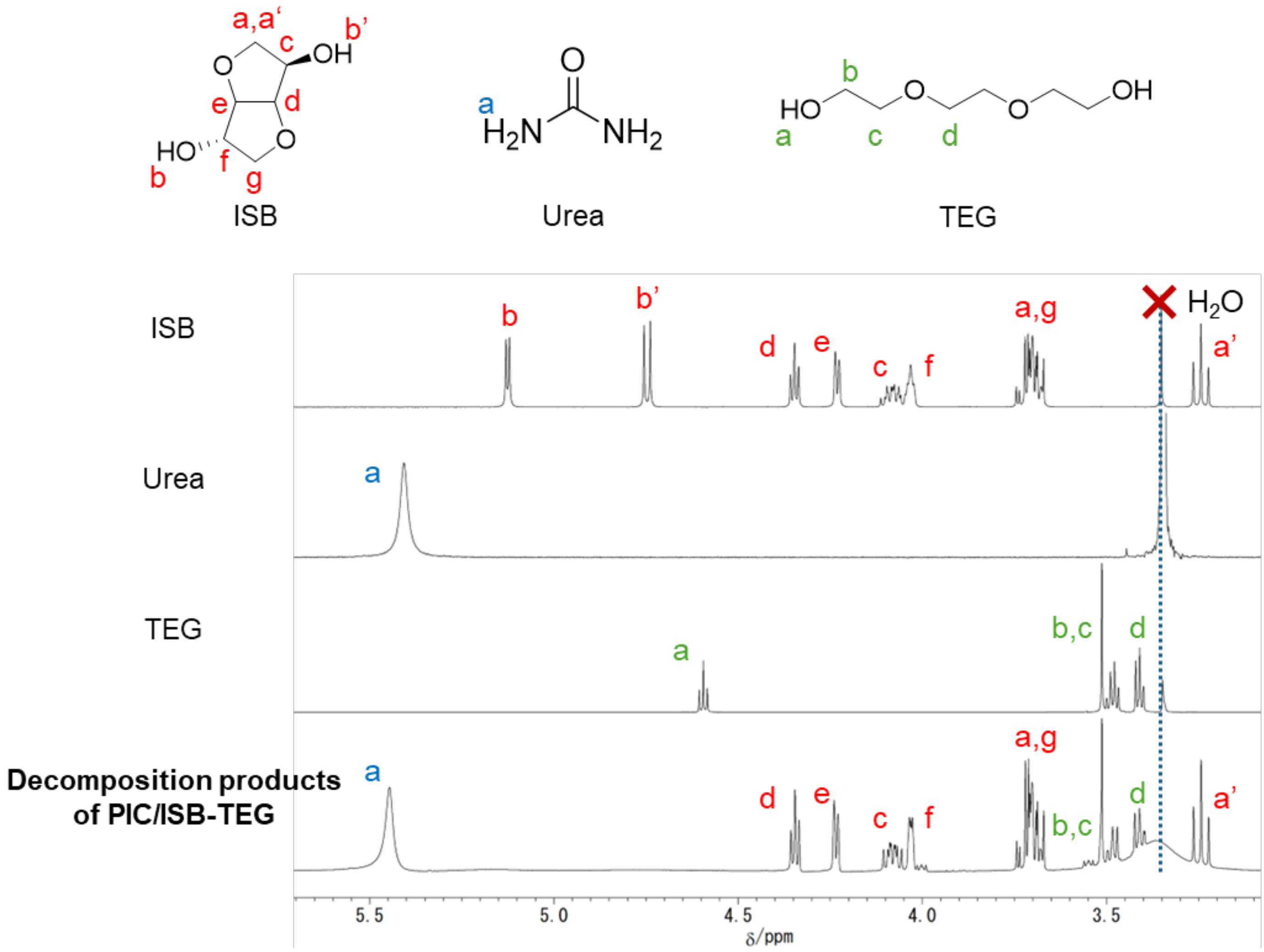

Figure S3. Partial ¹H NMR spectra of ISB, urea, TEG, and decomposition products (500 MHz, 298 K, DMSO-d_6).

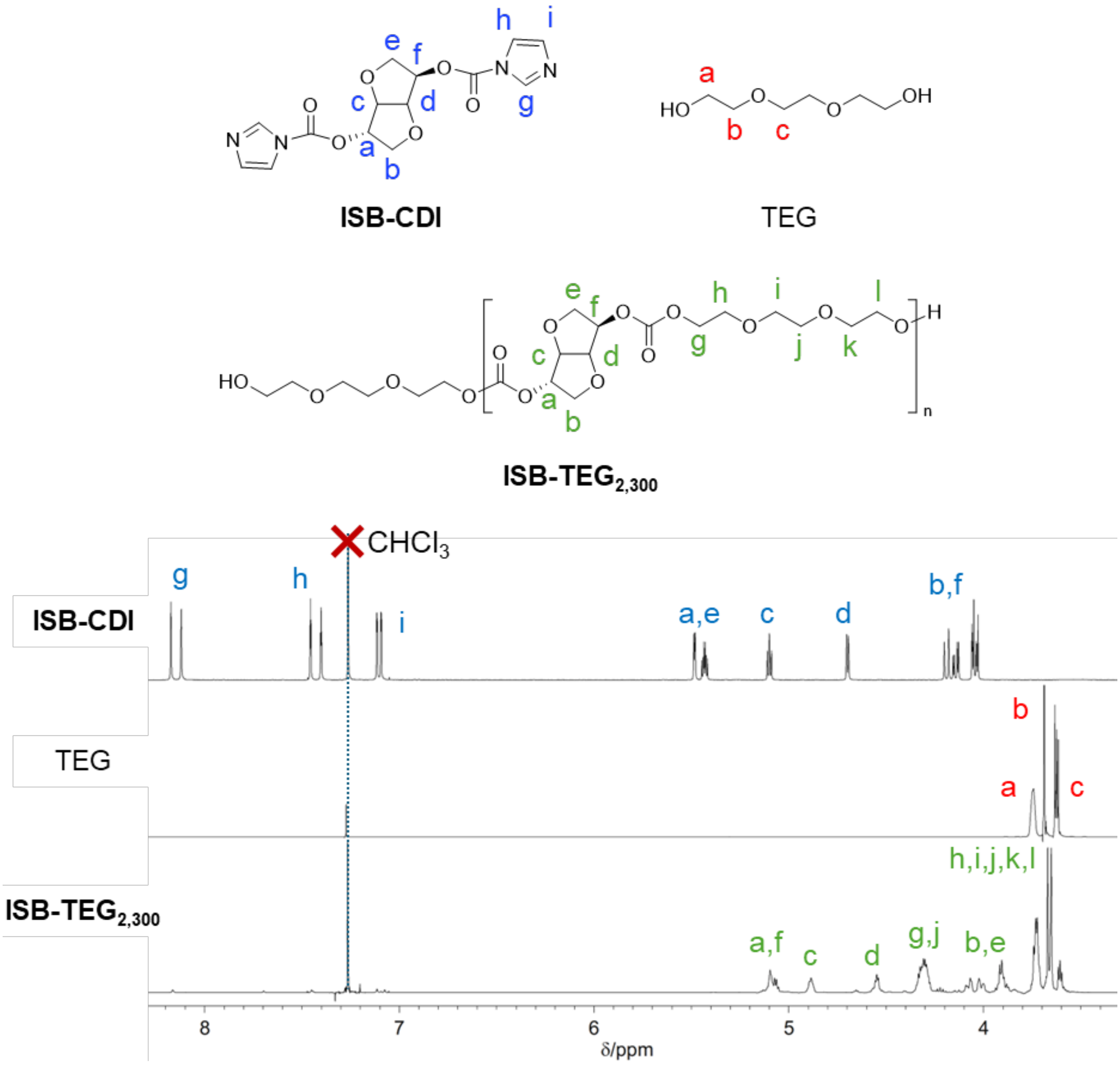

Figure S4. ^{1}H NMR spectra of **ISB-CDI**, TEG, **ISB-TEG$_{2,300}$** (500 MHz, 298 K, $CDCl_3$).

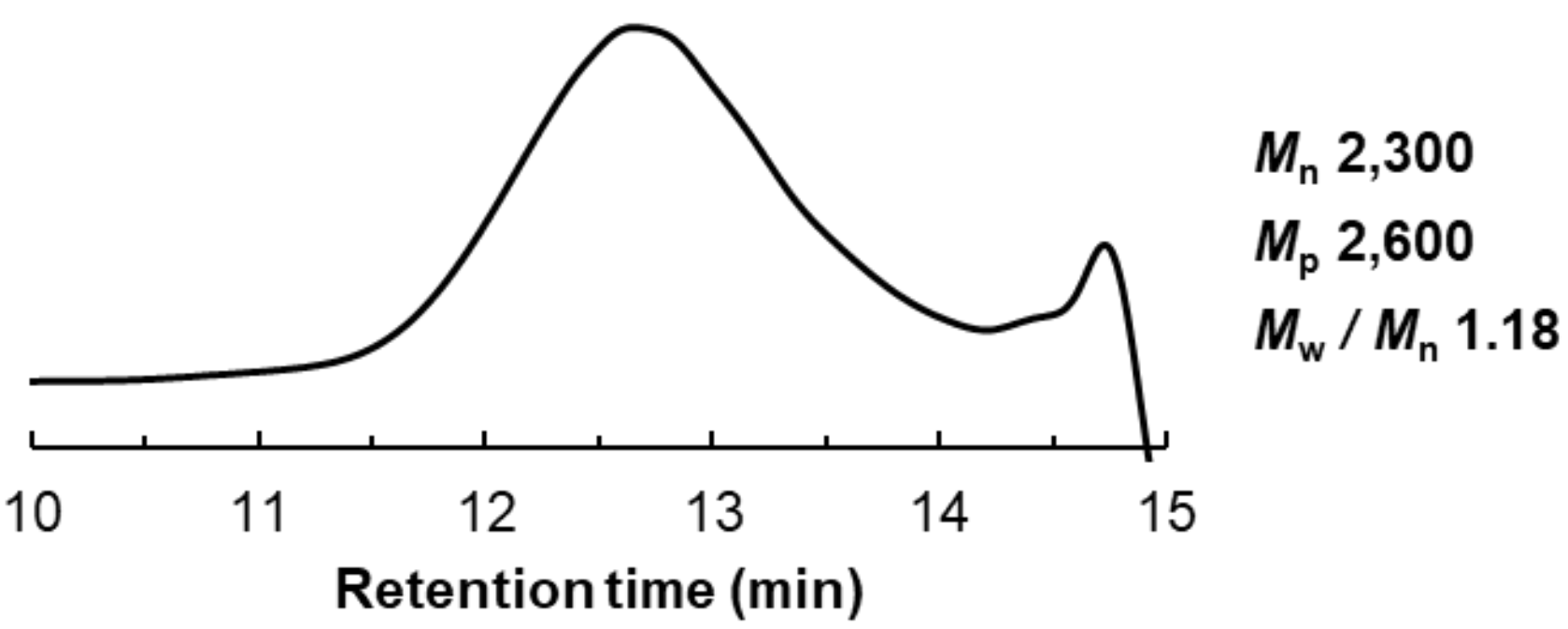

Figure S5. GPC profiles of **ISB-TEG$_{2,300}$** (eluent: DMF; detector: RI; standards: polystyrene).

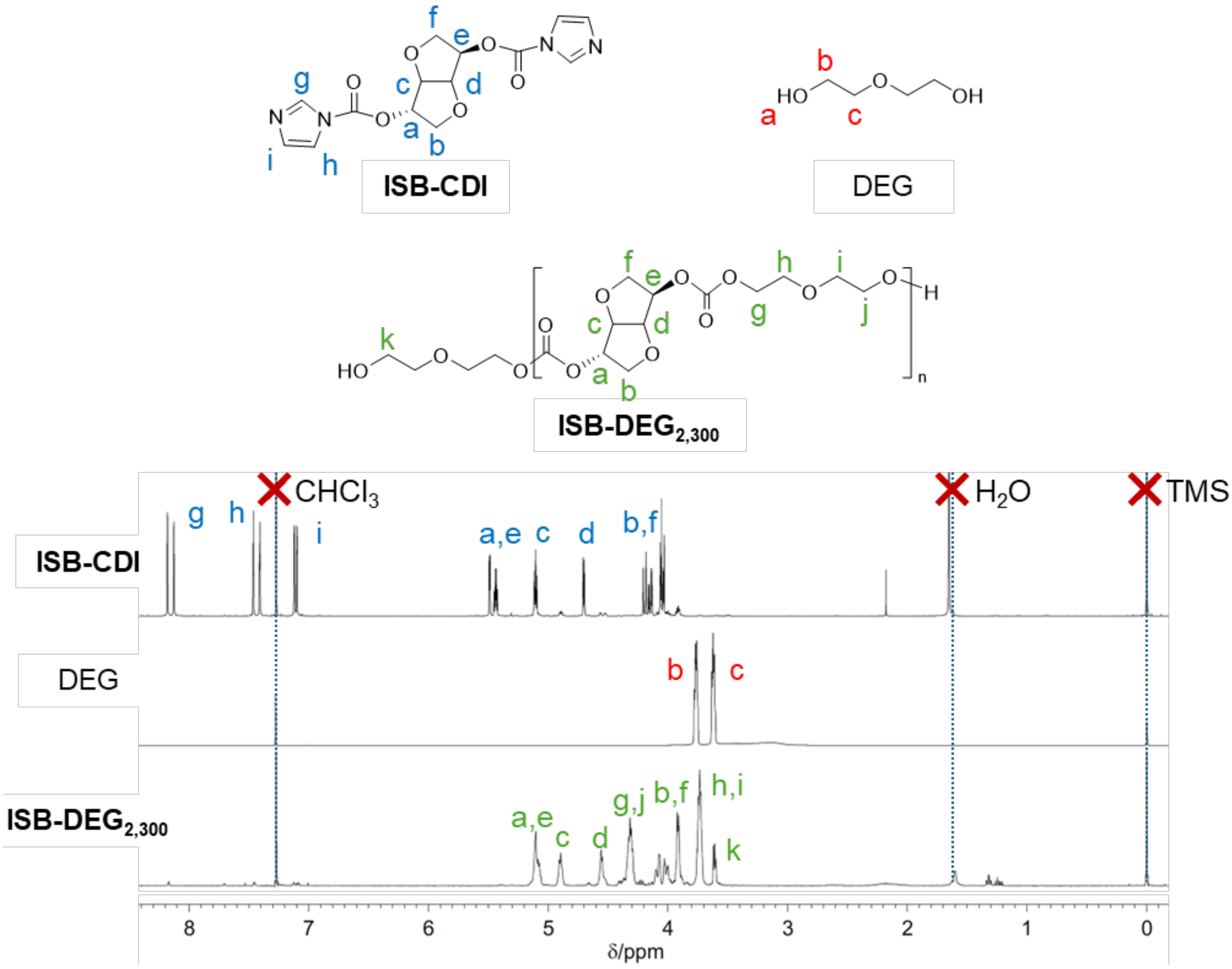

Figure S6. ^{1}H NMR spectra of **ISB-CDI**, DEG, **ISB-DEG$_{2,300}$** (500 MHz, 298 K, $CDCl_3$).

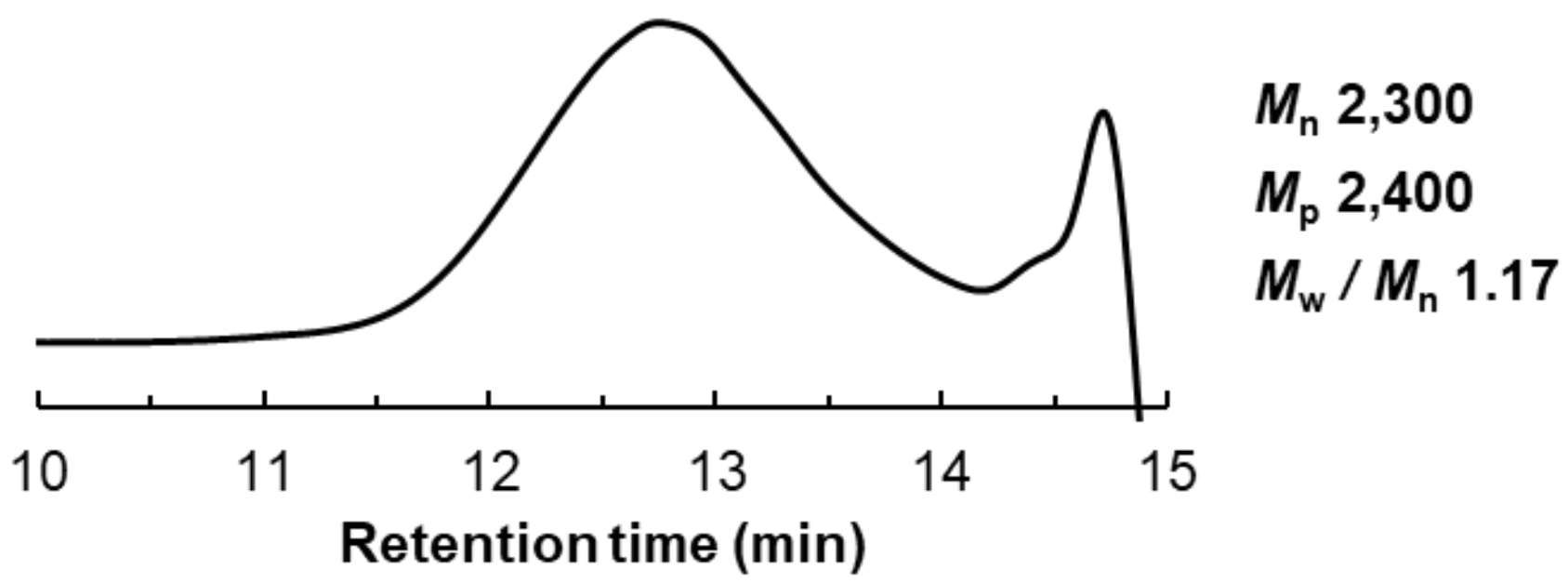

Figure S7. GPC profiles of **ISB-DEG$_{2,300}$** (eluent: DMF; detector: RI; standards: polystyrene).

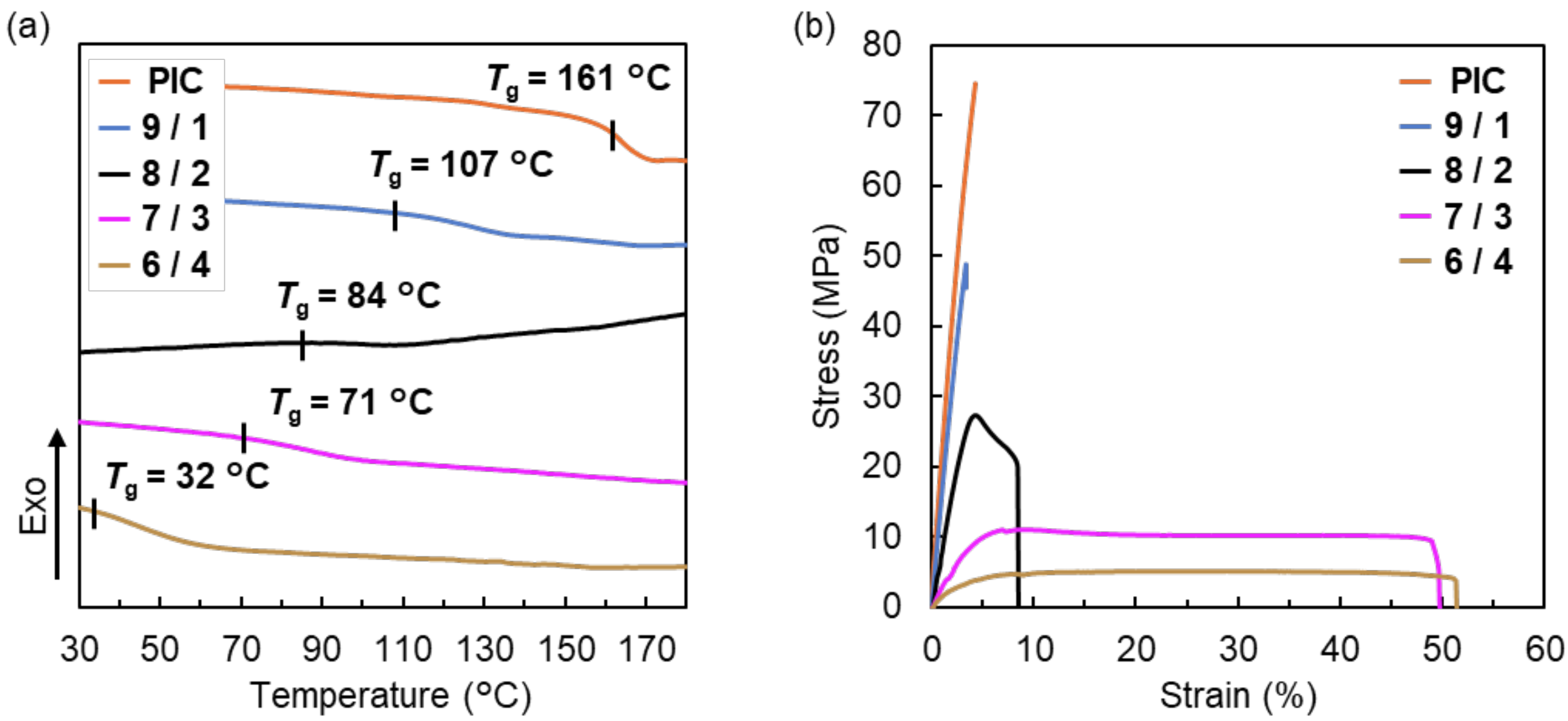

Figure S8. DSC curves of **PIC/ISB-DEG$_{2,300}$** samples of various ratios (T_g values are given for each polymer; 2nd heating; 10 °C /min) (c) Stress–strain curves for PIC and **PIC/ISB-DEG$_{2,300}$** samples of various ratios.

Table S1. Solubility of PIC.

Solvent or Plasticizer	δ_D	δ_P	δ_H	δ_t	Score[a]	*RED*
Toluene	18.00	1.40	2.00	18.16	0	1.84
Tetrahydrofuran	16.80	5.70	8.00	19.46	1	0.70
Dichloromethane	17.00	7.30	7.10	19.82	1	0.71
Methanol	14.70	12.30	22.30	29.41	0	1.83
Hexane	14.90	0.00	0.00	14.90	0	2.40
Ethyl acetate	15.80	5.30	7.20	18.15	0	1.22
Ethanol	15.80	8.80	19.40	26.52	0	1.62
DMF	17.40	13.70	11.30	24.86	1	0.94
Cyclooctane	16.60	0.10	0.10	16.60	0	2.47
Chloroform	17.8	3.1	5.7	18.95	1	0.86
Acetonitrile	15.30	18.00	6.10	24.40	1	0.87
Triphenyl phosphate	20.10	6.40	6.80	22.16	0	1.07
Triethylphosphate	16.70	11.40	9.20	22.21	0	0.77[b]
Triethyl citrate	16.50	4.90	12.00	20.98	0	1.44
Tributyl trimellitate	17.20	7.20	2.80	18.86	0	1.37
n-Butylbenzenesulfonamide	18.90	6.30	9.10	21.90	1	0.61
Dimethyl succinate	16.10	7.70	8.80	19.90	1	1.00
Dimethyl maleate	16.30	8.30	9.80	20.75	1	0.96
Dimethyl adipate	16.30	6.80	8.50	19.60	0	1.02
Dibutyl phthalate	17.80	8.60	4.10	20.19	0	1.16

[a]0 indicate insoluble, 1 indicates soluble.
[b]Worry out, which means that the HSPiP software prediction should be in the sphere, contrary to the experimental result.

Table S2. Solubility of **ISB-TEG**.

Plasticizer	δ_D	δ_P	δ_H	δ_t	score	RED
**ISB-TEG**	16.60	10.10	8.00	20.01	1	0.87

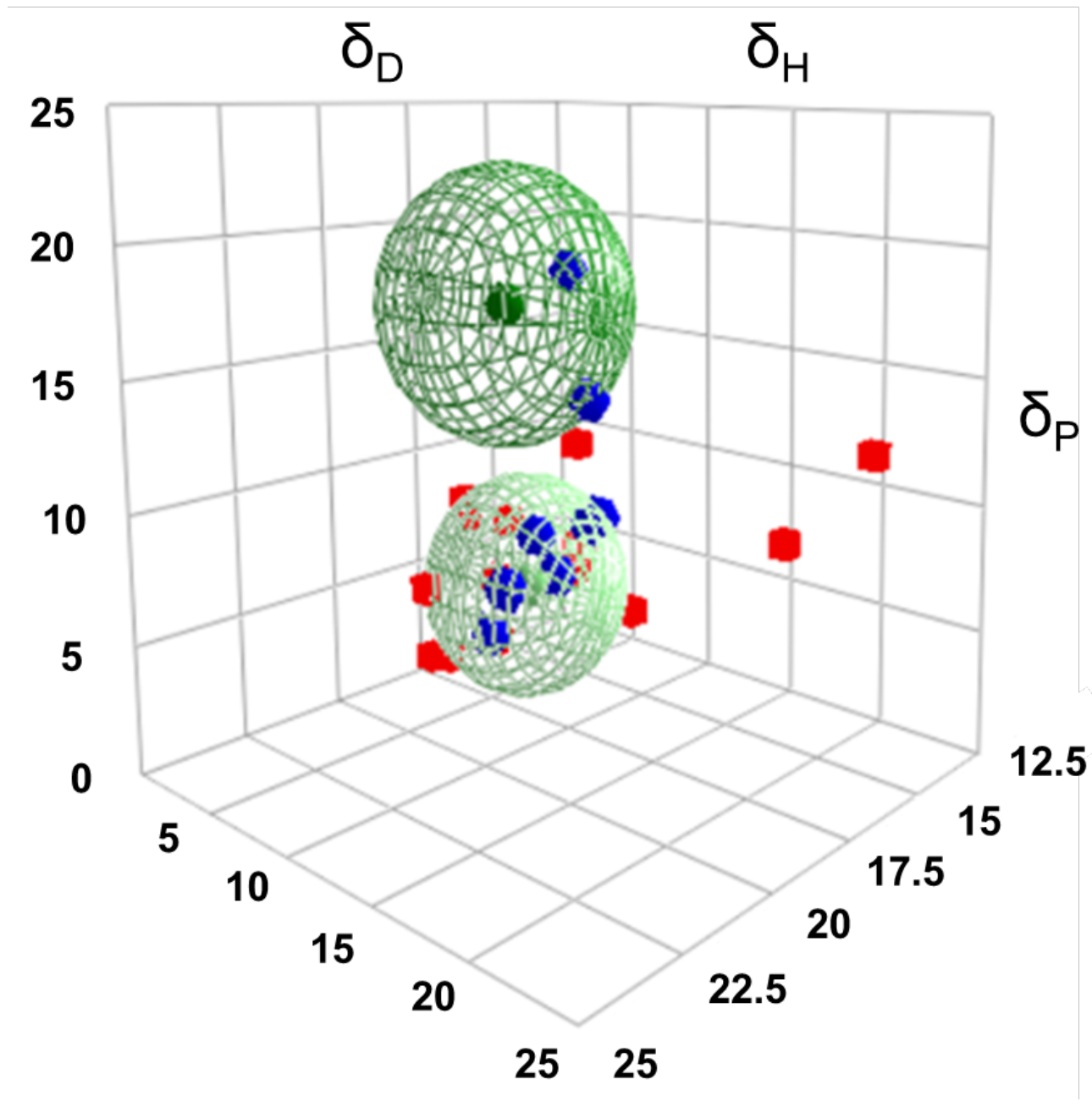

Figure S9. Double Hansen's spheres for PIC calculated with HSPiP. Red and blue dots indicate poor solvents and good solvents, respectively. Green HSP spheres indicate the regions of PIC categories.

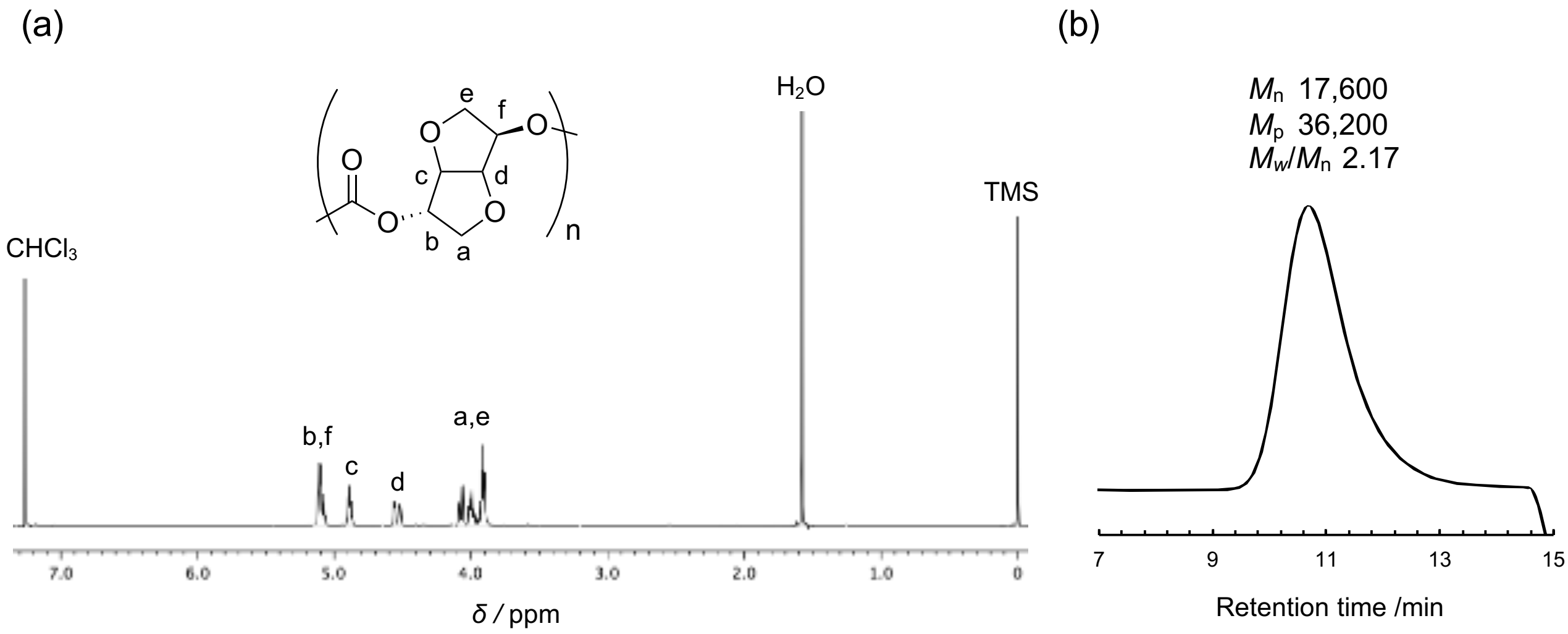

Figure S10. (a) ^{1}H NMR spectrum (500 MHz, 298 K, $CDCl_3$) and (b) GPC profile (eluent: DMF; detector: RI; standards: polystyrene) of PIC

4. Plant-growth-test

4.1. Plant-growth test of *Arabidopsis thaliana*[1]

Table S3. Fertilizers used in the plant-growth experiment.

Entry	Urea (300 mM)	Degradation products (300 mM)	Water with other minerals	Total volume
**1**	0 mL	0 mL	250 mL	250 mL
**2**	2.5 mL	0 mL	247.5 mL	250 mL
**3**	0 mL	2.5 mL	247.5 mL	250 mL

Table S4. Water with other minerals.

Entry	1	2	3
200×Pi	1.25 mL	1.25 mL	1.25 mL
200×Mg	1.25 mL	1.25 mL	1.25 mL
200×micro	1.25 mL	1.25 mL	1.25 mL
1M $CaCl_2$	500 μL	500 μL	500 μL
200×K_2SO_4 (300 mM)	1.25 mL	1.25 mL	1.25 mL
300 mM urea	0 mL	2.5 mL	0 mL
300 mM degradation products (ISB = 335 mM, urea = 300 mM, TEG = 95 mM)	0 mL	0 mL	2.5 mL

200×Pi: 350 mM NaH_2PO4, 350 mM Na_2HPO_4
200×Mg: 300 mM $MgSO_4$
200×micro: 200 µM $CuSO_4$, 6 mM H_3BO_3, 34 µM $(NH_4)_6Mo_7O_{24}$, 26 µM $CoCl_2$, 13.4 mM Na_2- EDTA, 1.72 mM $FeSO_4$, 2.06 mM $MnSO_4$, 200 µM $ZnSO_4$

4.2. Plant-growth test of Komatsuna[4]

Table S5. Soil properties.

Total carbon (g/kg)	Total nitrogen (g/kg)	NH_4-N (mg/kg)	NO_3-N (mg/kg)	pH	Cation Exchange capacity (cmol/kg)	Available phosphorus (P_2O_5 mg/kg)	Exchangeable potassium (K_2O mg/kg)	Soil texture
20.0	1.86	15	19	5.2	21.6	75	434	LiC

A pot culture test for Komatsuna (*Brassica rapa* var. *perviridis*) plants was conducted in Field Science Center, Tohoku University located in Osaki, Miyagi, Japan, using the degradation products obtained from the ammonolysis of **PIC/ISB-TEG** and commercially available urea. The ammonolysis of **PIC/ISB-TEG** or commercially available urea were applied to 2484 g of moist soil (1920 g as dry soil) at the rate of 0.50 g as nitrogen, respectively. A treatment without nitrogen source application (no nitrogen fertilizer treatment) was also set to clarify if the ammonia decomposition products of the ISB-based polymers with plasticizer were effective as nitrogen fertilizer. Each treatment was set in five replications. The soil was collected from plow layer (alluvial soil) of a field in Higashi Matsushima, Miyagi, Japan. Phosphorus and potassium fertilizers were applied to the soil in each treatment at the rate of 0.5 g as P_2O_5 and K_2O, respectively. Magnesium lime was also applied to the soil in each treatment at the rate of 2.0 g. The soils were well mixed with the above materials and fertilizers, and then put in 0.02 m^2 Wagner pots. Fifteen seeds of Komatsuna were sown in each pot. Then, soil moisture was adjusted to 60% of maximum water holding capacity by splaying deionized water. The pots were placed in a greenhouse using randomized block design. The temperature in the greenhouse was not controlled, and Komatsuna plants were grown there.

Throughout the entire growth period, irrigation with the deionized water was carried out daily to maintain soil moisture at 60% of maximum water holding capacity of the soil. The Komatsuna plants were thinned three times on 13 days after seeding (DAS), 16 DAS and 20 DAS to establish three plants per pot. The average air temperature in the greenhouse during the growth period was 26.2°C. The Komatsuna plants were harvested on 36 DAS. The fresh weight was measured immediately after harvesting, then oven dried at 70°C for 3 wk. Dried samples were weighed, then pulverized by a vibration mill (TI-100, CMT, Fukushima, Japan). The pulverized samples were digested by sulfuric acid with the occasional addition of hydrogen peroxide on a block digester (MB-2H-UII, Koike Precision Instruments, Itami, Japan), and then the digest was diluted to 50 mL. Nitrogen content of the digest solution was measured by the indophenol method (Scheiner 1976) with a spectrophotometer (U-2900, Hitachi High-Tech Corp., Tokyo).

Tukey's HSD multiple comparison test was carried out on the data of fresh weight and nitrogen uptake by Komatsuna plants. The statistical procedures were carried out using JMP Pro 17 (SAS Institute Japan, Tokyo).

5. Reference

1. Abe, T.; Takashima, R.; Kamiya, T.; Foong, C. P.; Numata, K.; Aoki, D.; Otsuka, H. Plastics to Fertilizers: Chemical Recycling of a Bio-Based Polycarbonate as a Fertilizer Source. *Green Chem.* **23**, 9030–9037 (2021).
2. Hult, D.; García-Gallego, S.; Ingverud, T.; Andrén, O. C. J.; Malkoch, M. Degradable High Tg Sugar-Derived Polycarbonates from Isosorbide and Dihydroxyacetone. *Polym. Chem.* **9**, 2238–2246 (2018).
3. Hansen, C. M. Hansen Solubility Parameters: A User's Handbook, 2nd ed.; CRC Press: Boca Raton, FL (2007).
4. Dora Scheiner. Determination of ammonia and Kjeldahl nitrogen by indophenol method. *Water Research*. **10**, 31-36 (1976).
